# Effect of Lanthanide Ions and Triazole Ligands on the Molecular Properties, Spectroscopy and Pharmacological Activity

**DOI:** 10.3390/ijms25147964

**Published:** 2024-07-21

**Authors:** Mauricio Alcolea Palafox, Nataliya P. Belskaya, Lozan T. Todorov, Nadya G. Hristova-Avakoumova, Irena P. Kostova

**Affiliations:** 1 Departamento de Química Física, Facultad de Ciencias Químicas, Universidad Complutense, 28040 Madrid, Spain; 2 Department of Technology for Organic Synthesis, Ural Federal University, 19 Mira Str., Yekaterinburg 620012, Russia; n.p.belskaya@urfu.ru; 3 Department of Chemistry, Faculty of Pharmacy, Medical University—Sofia, 2 Dunav Str., 1000 Sofia, Bulgaria; irenakostova@yahoo.com; 4 Department of Medical Physics and Biophysics, Faculty of Medicine, Medical University—Sofia, 2 Zdrave Str., 1431 Sofia, Bulgaria; nhristova@medfac.mu-sofia.bg

**Keywords:** lanthanide, structural relationships, DFT methods, infrared, Raman, DPPH, ABTS

## Abstract

The effect of La, Ce, Pr and Nd ions on four Ln(ligand)_3_ complexes and at three DFT levels of calculation was analyzed. Four ligands were chosen, three of which were based on the 1,2,3-triazole ring. The DFT methods used were B3LYP, CAM-B3LYP and M06-2X. The relationships established were between the geometric parameters, atomic charges, HOMO-LUMO energies and other molecular properties. These comparisons and trends will facilitate the synthesis of new complexes by selecting the ligand and lanthanide ion best suited to the desired property of the complex. The experimental IR and Raman spectra of Ln(2b′)_3_ complexes where Ln = La, Ce, Pr, Nd, Sm, Gd, Dy, Ho and Er ions have been recorded and compared to know the effect of the lanthanide ion on the complex. The hydration in these complexes was also analyzed. Additionally, the effect of the type of coordination center on the ability of an Ln(ligand)_3_ complex to participate in electron exchange and hydrogen transfer was investigated using two in vitro model systems—DPPH and ABTS.

## 1. Introduction

Bioinorganic chemistry, involving transition metals coordinated with various ligands, appears to be a very active research field today [1,2]. Coordination compounds with lanthanide ions and organic ligands are increasingly being synthesized and studied today for their numerous applications, particularly in medicine use [3,4,5,6]. However, facing the new social challenges now requires complexes with improved properties. Their design and further synthesis is a difficult task, and for this purpose and to facilitate it, previous relationships between geometric parameters and molecular properties should be established with related compounds. 

Therefore, with a focus on the design of new lanthanide complexes with better properties, we selected several ligands and lanthanide ions to establish relationships and trends that can be extrapolated to other complexes and may help in this search. Among the ligands selected for these complexes, those with heterocyclic rings containing nitrogen, oxygen or sulphur atoms are the most commonly used. Of particular interest are those containing a triazole ring, especially the 1,2,3-triazole structure. This heterocyclic ring has excellent selectivity in targeting different biological pathways associated with breast cancer [7,8,9,10,11] and also as an antifungal agent [12]. It also has favorable properties such as hydrogen bonding potential, moderate dipole moment and appropriate water solubility. Four different ligands have been used, three with this 1,2,3-triazole ring and the largest, the 2b′ ligand, which has shown potential anticancer activity [13,14].

Among the lanthanide ions, La(III) was initially chosen because it is one of the most widely used ions today [15,16]. Ce, Pr and Nd ions were also selected because of their biological properties [17,18,19]. 

The main aim of the present work was to facilitate the synthesis of new lanthanide complexes with better properties under the following specific points: (i) To elucidate the molecular structure and lanthanide–ligand binding mode with four proposed ligands. (ii) To establish new relationships and trends between the geometric parameters, atomic charges, energies and other molecular properties with four lanthanide ions. (iii) To determine the best DFT method with these complexes. (iv) To compare the experimental IR and Raman spectra with different lanthanide ion complexes and, therefore, to find the effect of the lanthanide ion on the spectra. (v) To study the impact of the type of Ln coordination center on the ability of the Ln(ligand)_3_ complex to participate in single electron exchange (SET) and hydrogen atom transfer (HAT) reactions.

## 2. Results and Discussion

### 2.1. Molecular Structure

**Figure 1:**
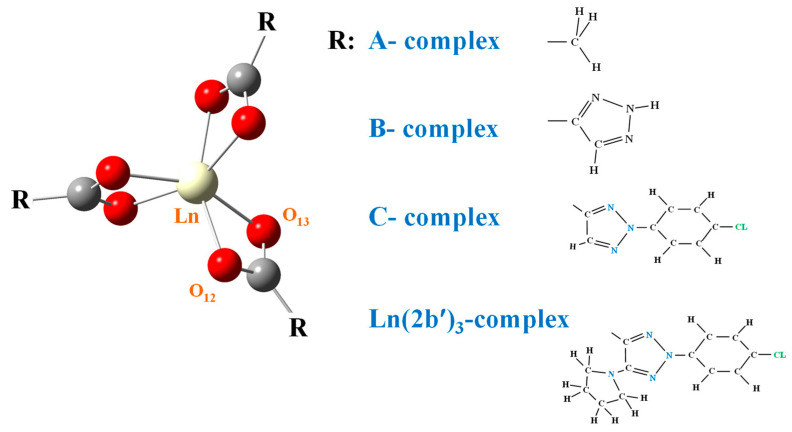
Schematic representation of the four Ln(III) complexes where R corresponds to methyl (A-complex); 1,2,3-triazole-4- (B-complex); 2-(4-chlorophenyl)-2*H*-1,2,3-triazole-4- (C-complex); and 2-(4-chlorophenyl)-5-(pyrrolidin-1-yl)-2*H*-1,2,3-triazole-4- (2b′ ligand) (Ln(2b′)_3_ complex).

In the complexes studied in the present work, Ln(ligand)_3_, where Ln = La, Ce, Pr and Nd, the lanthanide(III) ions are coordinated to the organic ligands via the oxygen atoms of a carboxylate group, which gives great flexibility to the structure. This type of coordination arrangement also occurs in complexes with other carboxylic acid derivatives, and it has previously been studied in a La-complex [15]. These carboxylate ligands are shown in Figure 1, which goes from the simplest one, where R is the methyl group (A-complex), to the largest size one with 2b′ (Ln(2b′)_3_–complex). 

**Figure 2:**
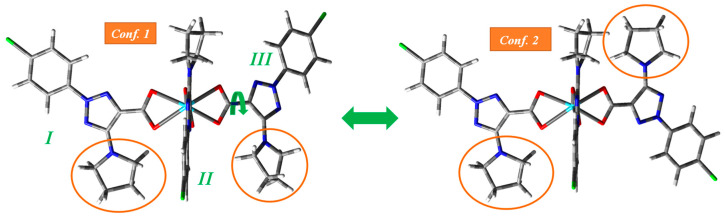
Two conformations found by rotation around the carboxylate group. The italics numbers in green color corresponds to the three 2b′ ligands of the complex.

All complexes were fully optimized with the B3LYP [20], CAM-B3LYP [21] and M06-2X [22] methods. In the case of the Ln(2b′)_3_ complex, two conformers were optimized by rotation around the carboxylate group, Figure 2, but only the most stable one (conformer 2) was considered in the tables and figures of the present work. Conformer 1 corresponds to an arrangement of the three ligands, pointed as *I* to *III* in this figure, in which they have the same orientation, whereas, in conformer 2, one of the ligands (ligand *III* in Figure 2) appears rotated 180° to the opposite side. Due to a lower steric repulsion of the pyrrolidine rings in this conformer 2, it is more stable than conformer 1. The energy difference between the two conformers was calculated to be about 20–30 kJ/mol, depending on the DFT method used.

**Figure 3:**
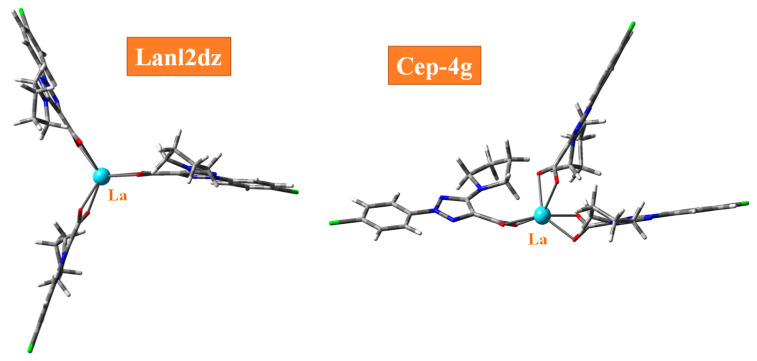
Optimized structure of the La(2b′)_3_ complex by the CAM-B3LYP method and the Lanl2dz and Cep-4g basis set.

The spatial arrangement of the ligands in these complexes depends on the theoretical method, the basis set used and the lanthanide atom. Therefore, for the La(III) ion and in the La(2b′)_3_ complex, the arrangement appears almost symmetric by CAM-B3LYP and M06-2X methods and using the Lanl2dz basis set, while it is deformed with two ligands close in packing form with the Cep-4g basis set (Figure 3). However, this last basis set is available in the Gaussian-16 program package in all lanthanum ions studied here. In these complexes, the Cep-121g basis set led to almost the same result as with Cep-4g, and because it requires more computer time, it was not used further. The Lanl2mb basis set also leads to this deformed packing form. The B3LYP method, without dispersion correction, leads to a deformed complex form, even using the Lanl2dz basis set. The dispersion forces appear to stabilize the ligands in the complex to a symmetrical orientation. The lanthanide atom also has a strong influence on the special arrangement of the ligands, as shown, for example, in Figure S1 (Supplementary Material section) for Pr(III) and Nd(III) ions in different orientations. This is due to the great flexibility of the carboxylate group, as well as the different atomic charges of the lanthanum ion and its distribution on the surrounding atoms. 

A summary of some selected optimized bond lengths of these complexes at three DFT levels is given in Table 1, while a larger list of calculated values is collected in Table S1 (Supplementary Material section), including bond angles and torsional angles between the ligands. Because of the different distributions of the ligands, small differences are expected in their geometric parameters, and therefore, for a systematic comparison of the values, only those of ligand *I* were included (Figure 2).

**Figure 4:**
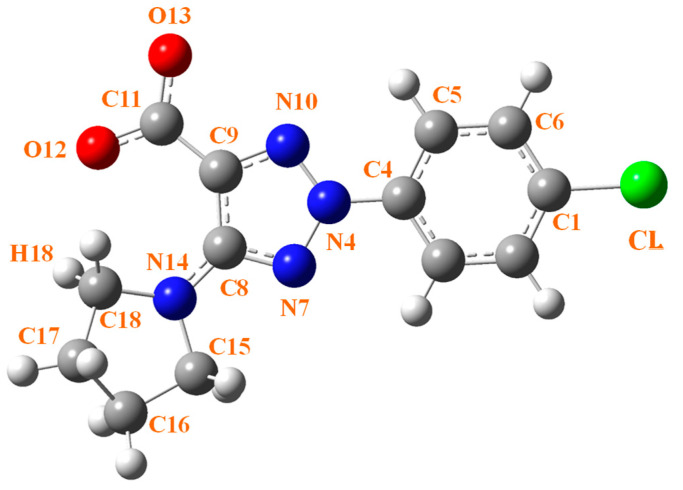
Labeling of the atoms in the 2b′ ligand of the complex.

The labeling of the atoms is shown in Figure 4 for the 2b′ ligand, as reported by Safronov et al. [13], and this notation has also been used for the remaining complexes. 

Compared to the B-complex, the planarity of the triazole ring is only slightly affected by the aryl substituent and by the pyrrolidine ring in all lanthanide complexes and at the three DFT levels used. As expected, the complete planarity of the aryl substituent is not affected by the Ln(III) ion of the complex. However, the pyrrolidine substituent is non-planar and has a noticeable non-coplanarity with the triazole ring plane, and this non-coplanarity value changes slightly depending on the lanthanide ion. This can be explained by the different atomic charge distribution around the lanthanide ions acting on the carboxylate oxygens, one of which is O12, intramolecularly H-bonded to the H18 hydrogen of the pyrrolidine ring. 

**Figure 5:**
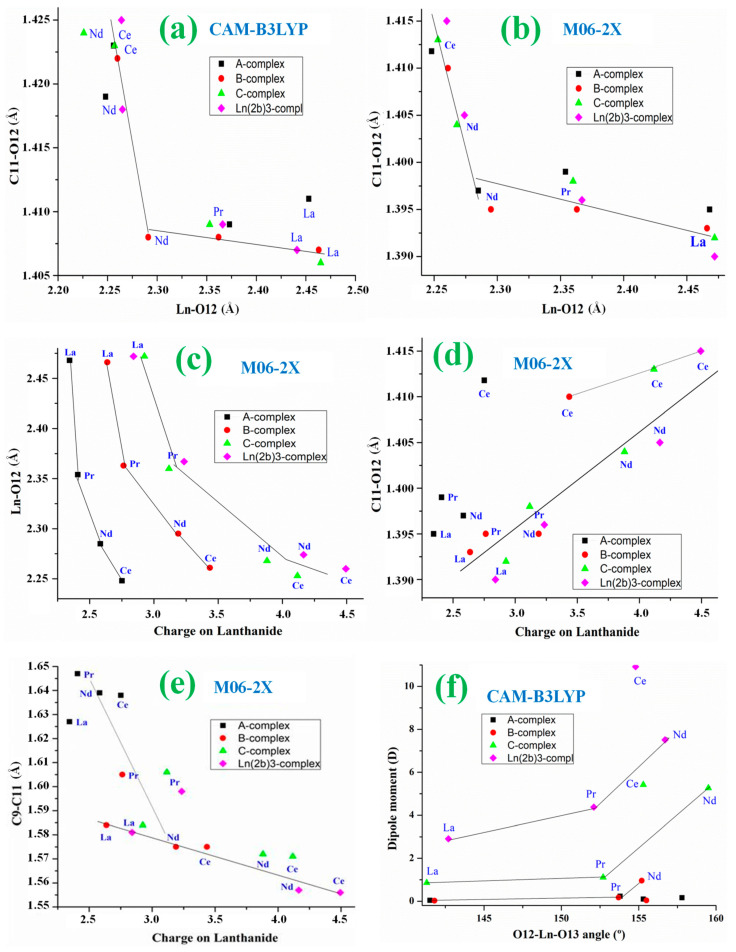
Relationships are established with the bond lengths and angles: (**a**,**b**) Between the Ln-O12 and C11-O12 bond lengths by CAM-B3LYP and M06-2X methods. (**c**) Between the Lanthanide charge and Ln-O12 bond length. (**d**) Between lanthanide charge and C11-O12 bond length. (**e**) Between lanthanide charge and C9-C11 bond length. (**f**) Between the O12-Ln-O13 angle and dipole moment of the complex.

**Figure 6:**
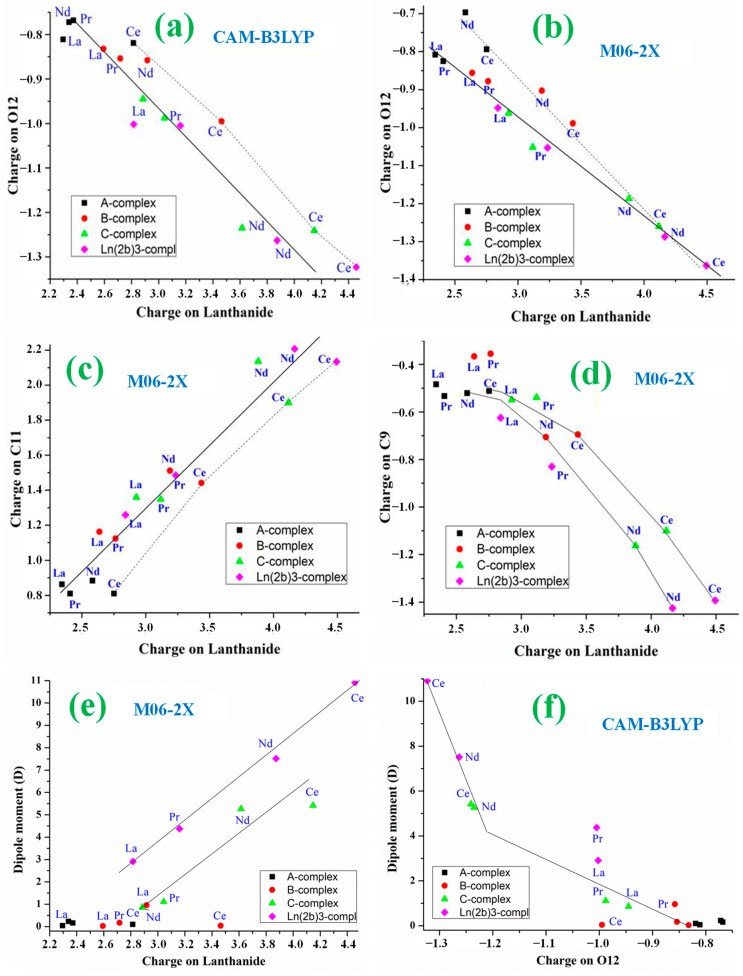
Relationships established with the atomic charges: (**a**,**b**) Between lanthanide charge and charge on O12 by CAM-B3LYP and M06-2X. (**c**) Between the lanthanide charge and charge on the C11 atom. (**d**) Between lanthanide charge and C9 charge. (**e**) Between the lanthanide charge and dipole moment of the complex. (**f**) Between the atomic charge on O12 and the dipole moment of the complex.

Comparing the values of this Table 1, the following can be noted:

(i) The Ln-O12 bond length value depends mainly on the nature of the Ln ion, with the longest value for complexes with La(III) ion and the shortest one for complexes with Ce(III) ion. Close values appear for those with Ce and Nd ions. Similar features are obtained for the Ln-O13 bond length.
(ii) With the CAM-B3LYP method, the Ln-O12 bond length seems to be little affected by the ligand size in the complexes with the Ce(III) ion with values in the 2.256–2.264 Å range, but it seems to be strongly affected in the complexes with Nd(III) ion, with values in the 2.226–2.291 Å range. A strong effect with Nd ion is also observed with the M06-2X method, but the range is slightly shorter at 2.268–2.295 Å.
(iii) As expected, the effect of the Ln ion nature is significantly reduced as the bond length increases. Therefore, the C11-O12 bond length varies in the short 1.406–1.425 Å range by CAM-B3LYP and 1.390–1.415 Å by M06-2X, with the shortest value in La complexes and the largest one in Ce complexes.
(iv) The C11-O12 bond length seems to be less affected by the ligand effect than the Ln-O12 bond. Thus, the values appear in the shortest 1.422–1.425 Å range for the Ce complexes and in the 1.408–1.424 Å range for the Nd complexes.
(v) Lengthening of the Ln-O12 bond leads, as expected, to a shortening of the C11-O12 bond lengths, but this shortening is large in the Nd complexes and smaller in the other lanthanide complexes. A linear relationship between the Ln-O12 and C11-O12 bond lengths can be established in the Ce and Nd complexes and in the La and Pr complexes by both CAM-B3LYP (Figure 5a) and M06–2X (Figure 5b).
(vi) The C9-C11 bond length is noticeably affected by both Ln charge and ligand bonded. Therefore, its value varies in the long 1.556–1.647 Å range by M06-2X and similar long-range by CAM-B3LYP, 1.562–1.656 Å, indicating a large flexibility of this C9-C11 bond for adapting new bonded ligands.
(vii) In the smallest A-complexes, the value of the O12-Ln-O13 angle has no effect on the dipole moment of the complex. However, for larger ligands, it has a noticeable effect. Therefore, as the size of the ligand is increased, the dipole moment is increased (Figure 5f). This effect is further enhanced by increasing the positive charge on the Ln ion. Thus, the Ce(2b′)_3_ complex has a huge dipole moment, followed by the Nd(2b′)_3_ complex. The large dipole moment value in these complexes indicates that they may have a large water solubility, which could facilitate their biomedical use. By contrast, La and Pr complexes with small dipole moment values are not useful for this purpose. The large increment in the dipole moment with the increase in the Ln charge can be explained by the opening of the O12-Ln-O13 angle and a rotation of the ligands to avoid steric interactions. A relationship between this angle and the dipole moment can be established for each type of complex (Figure 5f). A good linear relationship can be established between the dipole moment and the Ln charge for the C-complexes and the Ln(2b′)_3_ complexes, Figure 6e. The relationship seems to be worse for the O12 charge, Figure 6f.
(viii) The C9–C11···C11′–C9′ torsional angle between the ligands in C- and Ln(2b′)_3_ complexes also has a noticeable effect on the dipole moment. An increase in its value in Ln(2b′)_3_ complexes seems to be well linearly related to a remarkable reduction in the dipole moment by both CAM-B3LYP and M06-2X methods (Figure S2). La-complexes do not follow this relationship.

### 2.2. Atomic Charges

Some selected calculated APT (Atomic Polar Tensor) charges in all complexes and three DFT methods are shown in Table 2, while a larger collection of values with other atoms and different distributions of them is shown in Table S2. Although the NBO charges are more accurate than APT charges, unfortunately, they are not available in Gaussian-16 for the Ce, Pr and Nd ions. In order to know the effect of these charges on the dipole moment of the complex, their values have also been included in Table 2. The charge value of the lanthanide ion has a strong influence on the other atomic charges and on the geometric parameters of the complex, especially on those around the neighboring atoms. 

The following observations can be made by comparing the values obtained in Table 2:

(i) The atomic charge of Ce and Nd ions is significantly higher than that of Pr and La ions, with the value of La ion being the lowest in all their complexes. These lowest values on La complexes lead to the longest La-O12 and La-O13 bond lengths. By contrast, the highest values on Ce complexes lead to the shortest La-O12 and La-O13 bonds. A clear relationship between Ln charge and Ln-O12 bond length can be established for each type of complex; Figure 5c by M06-2X and Figure S3a by CAM-B3LYP.
(ii) The complexity of the ligand has a noticeable effect on the positive Ln charge. As the ligand becomes larger and more complex, the Ln charge increases up to a level, such as in C-complex, where further increases in ligand size have little effect on the Ln charge (Figure 5c). The effect of the ligand size is noticeably higher for Ln ions with a large positive charge, such as the Ce ion than for those with a small charge, the La ion. This can be explained by the ability of the Ln ions to donate electrons to the ligands, and the amount of electronic charge to be transferred is increased with large ligands because it can be more easily distributed on the molecular structure.
(iii) An increase in Ln charge appears to be somewhat linearly related to a lengthening of the C11-O12 bond (Figure 5d). The values in A-complex do not appear to follow this relationship, nor do the Ce values, which follow another similar linear relationship. The CAM-B3LYP and B3LYP relationships appear worse and have therefore been included as Supplementary Material (Figure S3).
(iv) The increase in the positive Ln charge is well linear and related to an increase in the negative charge on O12 by both CAM-B3LYP and M06-2X methods, in Figure 6a and Figure 6b, respectively. This relationship is also well established with the atomic charge on O13 (Figure S4) as well as by B3LYP. The complexes with the Ce ion follow a similar linear relationship. This withdrawal of electronic density by the carboxylate oxygen atoms comes from both the Ln ion and the C11 atom, increasing its positive charge. A good linear relationship with the C11 atom has also been obtained with the M06-2X method (Figure 6c) and with CAM-B3LYP (Figure S4f).
(v) Since the Ln charge value affects the Ln-O12, Ln-O13 and C11-O12 bond lengths, as well as the atomic charges O12, O13 and C11, the C9-C11 bond length also appears to be affected by the Ln charge with a somewhat linear relationship (Figure 5e). The values in A-complex do not seem to follow this relationship.
(vi) The nitrogen atoms N4 and N7 have a negative charge, while the charge in N10 is slightly positive, mainly due to the withdrawal of electronic charge from the C9 atom. A relationship between this C9 charge and the Ln charge can be established, especially for the Ce and Nd complexes; Figure 6d with the M06-2X method and Figure S5b with CAM-B3LYP. Relationships were not observed by B3LYP (Figure S5a) nor with the N4 atomic charge (Figure S5c,f).

### 2.3. Molecular Properties

Several selected thermodynamic parameters have been determined by three DFT methods in the four complexes under study, and their values are included in Table 3. Additional parameters are collected in Table S3 in different arrangements. The energy values of the HOMO (highest occupied molecular orbital) and LUMO (lowest unoccupied molecular orbital) frontier orbitals were used to calculate the energy gap (Eg = E_LUMO_ − E_HOMO_), which is an important property of the complexes and it will help to determine their chemical reactivity and kinetic stability. The HOMO and LUMO energies have also been used to calculate several global chemical reactivity descriptors [23,24] by means of the following formulae:

IP = −E_HOMO_

EA = −E_LUMO_

χ = −(E_HOMO_ + E_LUMO_)/2

η = (E_LUMO_ − E_HOMO_)/2

S = ½ η

**Figure 7:**
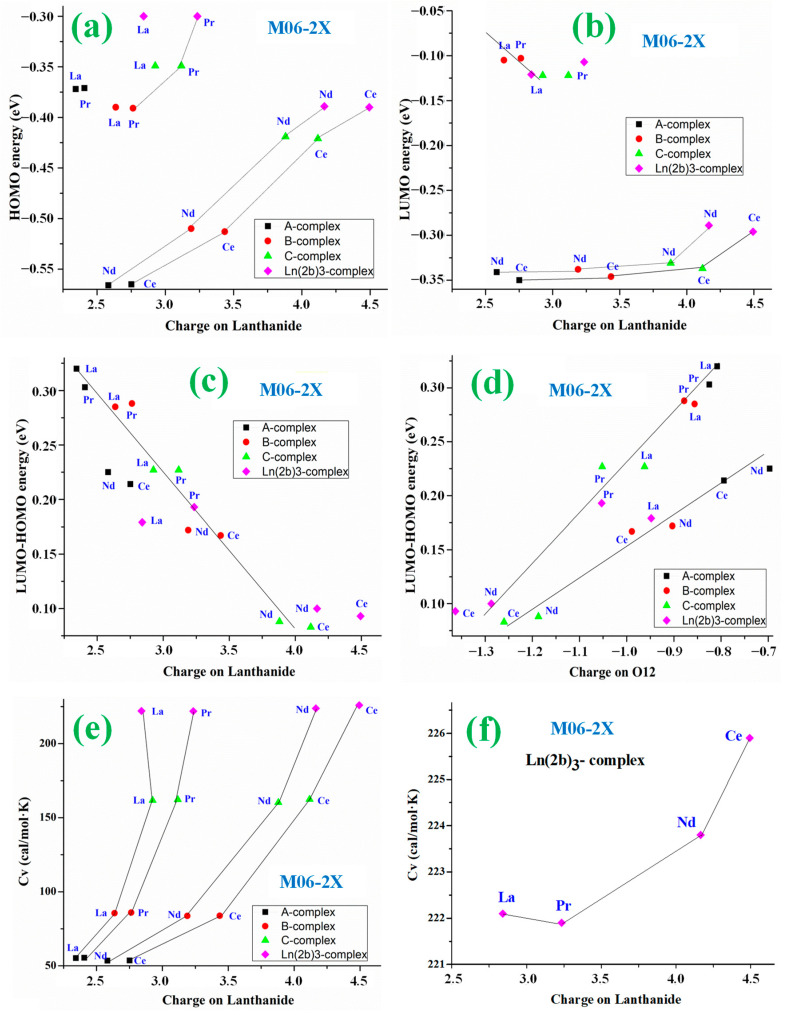
Relationships established at the M06-2X level with the molecular properties: (**a**) Between lanthanide charge and HOMO energy. (**b**) Between the charge on O12 and LUMO energy. (**c**,**d**) Between the LUMO-HOMO energy and the lanthanide/O12 charges, respectively. (**e**,**f**) Between lanthanide charge and capacity at constant volume in all complexes and in Ln(2b′)_3_ complex, respectively.

A comparison of the values collected in Table 3 and Table S3 allows the following observations to be made:

(i) In absolute values, the HOMO and LUMO energy values in La and Pr complexes are significantly lower than in Ce and Nd complexes. Increasing the ligand size noticeably decreases the HOMO energy, especially in Ce and Nd complexes. In these complexes, an almost linear relationship related to the Ln charge can be observed (Figure 7a) by M06-2X and (Figure S6c) by CAM-B3LYP.
(ii) The ionization potential (IP) values are the lowest for the Ln(2b′)_3_ complexes, corresponding to the largest reactivity of these complexes due to their large size, while the IP values are the highest for the A-complexes (Table S3).
(iii) The LUMO energy value does not change significantly with increasing Ln charge increase in Ce and Nd complexes, except for Ce(2b′)_3_ and Nd(2b′)_3_ complexes, while it is slightly increased in La and Pr complexes, by M06-2X in Figure 7b and by CAM-B3LYP in Figure S6d.
(iv) Although band gaps calculated at the DFT level are typically underestimated, a high energy gap Eg is calculated in A- and B-complexes, especially in La and Pr complexes, indicating that these complexes are poorly polarizable, with low chemical reactivity and high kinetic stability. In contrast, C- and Ln(2b′)_3_ complexes with Ce and Nd ions have very low Eg values by all DFT methods, indicating high chemical reactivity and low excitation energies to the manifold of excited states of these complexes. A somewhat linear relationship of the decrease in Eg with the increment of Ln charge has been established by M06-2X in Figure 7c and by CAM-B3LYP in Figure S7a.
(v) A similar relationship but with the O12 charge appears plotted in Figure 7d, where an increase in the negative charge of O12 leads to a low value of Eg. Two linear relationships can be established, one with the Ce and Nd complexes and another with the La and Pr complexes.

(vi) Chemical hardness (η) and global softness (S) indicate the resistance of a system to a change in its number of electrons. For simplicity, Table 3 only includes the values of *S*, while Table S3 collects both *S* and η. The lowest values of η and *S* correspond to C- and Ln(2b′)_3_ complexes with Ce and Nd ions, and because of their low values, these complexes can be called soft, indicating a small gap and an electron density that can easily change.
(vii) The C_v_ values strongly depend on the ligand size, as expected. The CAM-B3LYP method calculates them slightly higher than M06-2X, around 1–4 cal/mol·K in Ln(2b′)_3_ complex. The Ln ion has little effect on this value, with the highest value in Ce(2b′)_3_ complex, 225.9 cal/mol·K by M06-2X, and the lowest in Pr(2b′)_3_ complex, 221.9 cal/mol·K (Figure 7f). A similar small difference has also been calculated by CAM-B3LYP (Table 3). A clear relationship between the increment of Cv value and the increase in the Ln charge for each Ln complex has been plotted in Figure 7e by M06-2X and in Figure S7d by CAM-B3LYP.
(viii) The entropy (S) values depend on the ligand size and the symmetry of the complex. Therefore, it has the highest value in the La(2b′)_3_ complex determined by B3LYP due to its lowest symmetry obtained by this method. Due to the large flexibility of the carboxylate group and the different arrangement of the ligands in the complex, the S value shows slight variations among the complexes (Table S3).
(ix) The rotational constants in the three directions (A, B, C) have different values, with one being the highest (A-axis) in all complexes and one being the lowest (C-axis). As the ligands are most flexible in the A-complexes, the values of the rotational constant are the largest in these complexes, whereas the calculated smallest values appear in the Ln(2b′)_3_ complexes.

### 2.4. Infrared and Raman Spectra

**Figure 8:**
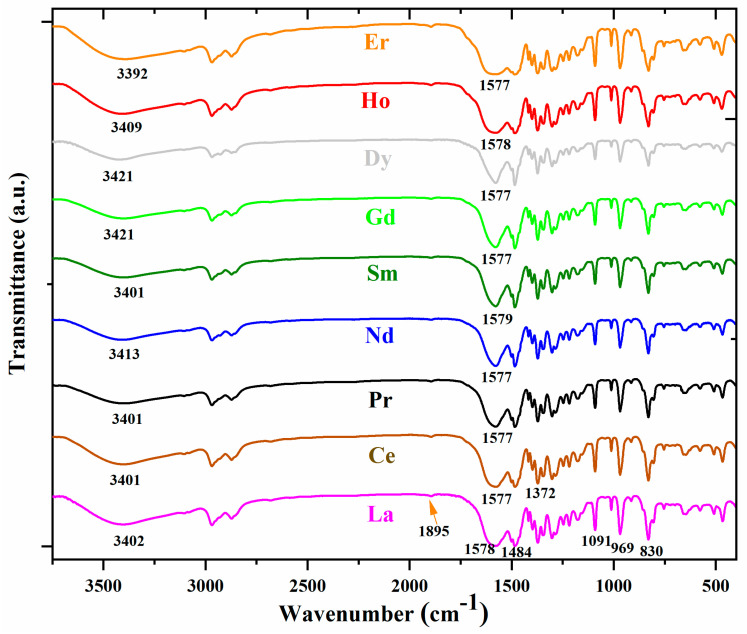
Comparison of the experimental IR spectra of the Ln(2b′)_3_ complexes with Ln = La, Ce, Pr, Nd, Sm, Gd, Dy, Ho and Er in the 3750–400 cm^−1^ range.

**Figure 9:**
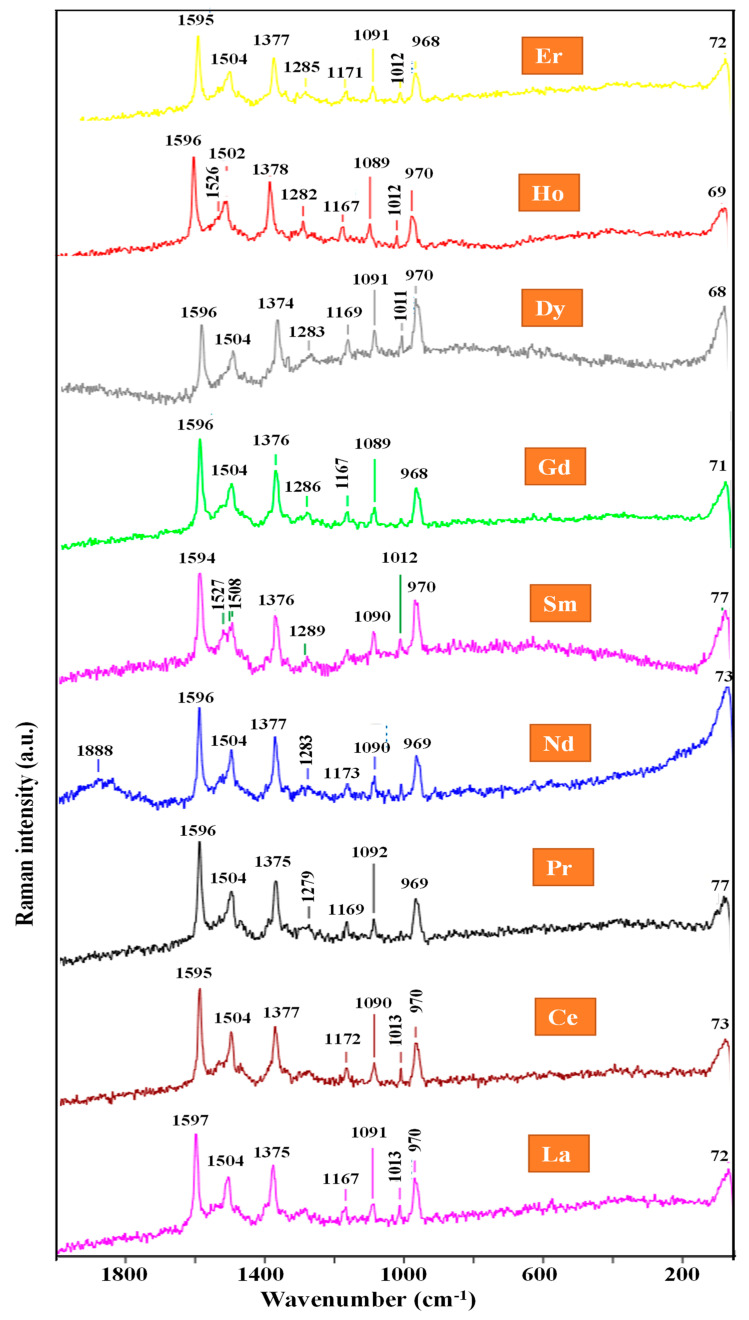
Comparison of the experimental Raman spectra of the Ln(2b′)_3_ complexes with Ln = La, Ce, Pr, Nd, Sm, Gd, Dy, Ho and Er in the 2000–50 cm^−1^ range.

In a previous study, the experimental IR and Raman spectra of La(2b′)_3_ and Ce(2b′)_3_ complexes were fully interpreted with the help of theoretical DFT calculations and accurate scaling procedures [15,25]. To know the effect of lanthanum ions in the IR and Raman spectra of Ln(2b′)_3_ complexes, the experimental spectra with a set of these ions Ln = La, Ce, Pr, Nd, Sm, Gd, Dy, Ho and Er have been recorded and compared in the present manuscript (Figure 8 and Figure 9). In these figures, the wavenumbers of the most intense IR/Raman bands are indicated, especially those related to the carboxylate COO group, which are expected to somewhat change with the Ln ion. These Ln complexes have been selected because of the important biological activities they are expected to have. For example, samarium complexes have antioxidant activities [26], and dysprosium complexes appear to have antitumor activities [27].

When comparing the IR spectra of Figure 8, the following main features are observed:

(i) The spectra appear very close in both IR intensity and wavenumber position of all bands. This means that the ligands are very little affected by the different Ln ions. Therefore, the characterization and assignment previously carried out for La(2b′)_3_ and Ce(2b′)_3_ complexes [15,25] can largely be applied to the bands of the remaining lanthanide Ln(2b′)_3_ complexes.
(ii) The very broad band at 3400 cm^−1^ corresponding to the O-H stretching ν(O-H) mode of the hydration water molecules H-bonded to the oxygen and nitrogen atoms of the ligands shows small shifts in its maximum in the different Ln complexes. Compared to the La complex, the highest blue-shift in its wavenumber up to 3421 cm^−1^ was observed in the Gd and Dy complexes, indicating that in them, the water molecules appear slightly less H-bonded to the ligands, while the highest red-shift up to 3392 cm^−1^ corresponds to the Er complex. In the latter case, the lowest ionic radius of Er seems to facilitate the entry of the hydration water molecules associated with the complex synthesis into enter inside of the carboxylate region and to have slightly stronger H-bonds.
(iii) The δ(O-H) in-plane bending mode of the hydrated water molecules appears to make a large contribution to the broadening of the broad and very strong experimental band centered at 1578 cm^−1^ and assigned to the stretching ν(C8-N14) + 8a, ν(CC) mode [15]. The lowest ionic radius of Ho and Er appears to increase the amount of H-bonded water molecules in these complexes. Therefore, the width of this band significantly increases in the spectra of these complexes.
(iv) A very weak band at ca. 1895 cm^−1^ appears in all spectra that, by theoretical calculations [15], can be assigned to water molecules strongly H-bonded and, therefore, with large O-H bond lengths. The very weak intensity indicates that very few water molecules are involved in this H-bond, and the very large red-shift in its wavenumber indicates that this H-bond should have highly positively (Ln) or negatively (oxygen) charged atoms.

The same comparison but with the Raman spectra of the Ln(2b′)_3_ complexes is shown in Figure 9. Due to the large background noise in the experimental spectra, which made it difficult to detect all the weak bands, the comparison was made only in the 2000–50 cm^−1^ region. Bands corresponding to water molecules do not appear in these spectra, making interpretation easier. The main effects observed in this comparison are as follows:

(i) The large similarity among the Raman spectra confirms what was observed in the IR spectra, where the ligands are very little affected by the different Ln ions.
(ii) A strong and broad band appears at 72 cm^−1^ in La complex, where the wavenumber varies in the 68−73 cm^−1^ range for each Ln complex. Small peaks at 77 and 96 cm^−1^ are also observed within this broad band. Its intensity is significantly enhanced in Sm, Dy and especially in Nd complexes. This band has been assigned to an out-of-plane vibrational mode of the lattice net, mainly involving the Ln ion. The width of this band and the peaks at 68, 72, 77 and 96 cm^−1^ can be interpreted by the different and close arrangement of the three ligands in the complex by the effect of the water molecules and the Ln ion.

(iii) Only in the spectrum of the Nd complex appears a broad and weak band centered at 1888 cm^−1^, and only in this spectrum is the broad band at 73 cm^−1^ noticeably enhanced. Because of these two characteristics, it is possible to relate them, and it may be due to the special and different environment in which the Raman spectrum of the sample was recorded.
(iv) The band at 1167 cm^−1^ in the La complex and assigned to the ν_s_(NNN) of the triazole ring, is the one with the higher wavenumber shift and intensity with the different Ln ions, although the shifts are small, around 6 cm^−1^. The bands at 1375 and 970 cm^−1^ are also assigned to this stretching mode and are strongly coupled with the ν(COO) mode, as well as with other ring modes.

### 2.5. Participation in HAT and SET

**Figure 10:**
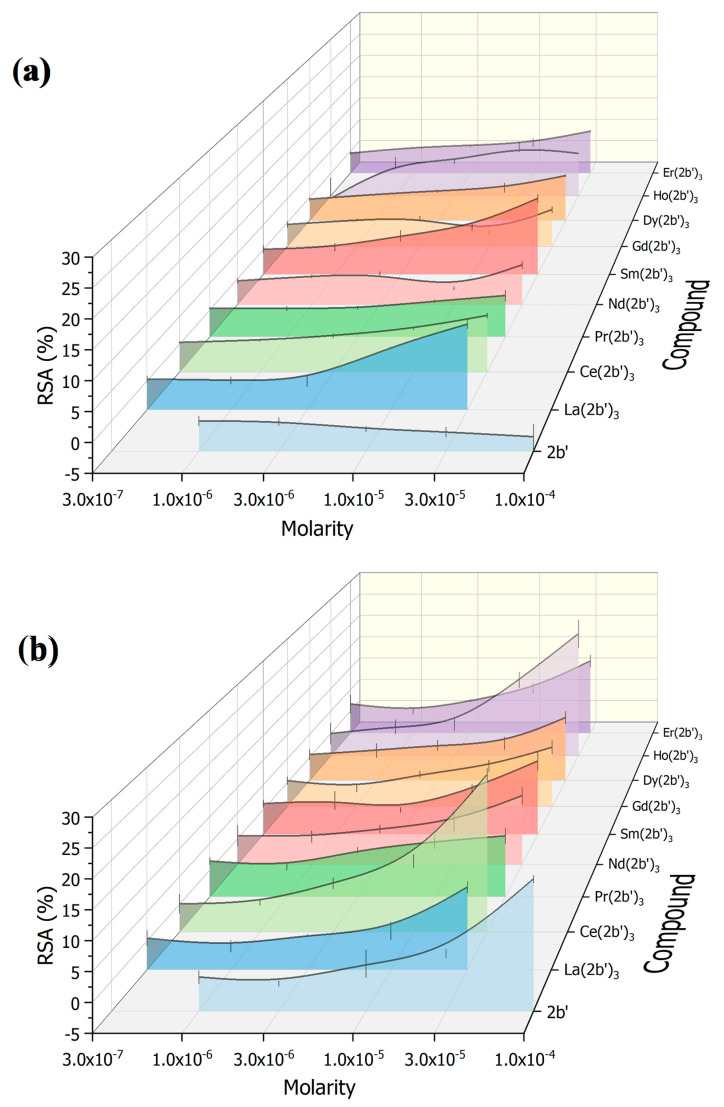
Impact of the ligand 2b′ and the various Ln(2b′)_3_ complexes on (**a**) the DPPH and (**b**) ABTS in vitro model systems. Data = mean ± stdev, N = 4.

In order to confirm what impact the type of Ln coordination center has on the reactivity of an Ln-1,2,3-triazole complex, the authors have conducted two types of in vitro assays involving the Ln(2b′)_3_ complexes:

(1) 2,2-diphenyl-1-picrylhydrazyl (DPPH) assay, involving the stable radical DPPH^●^. This radical helps to assess the ability of a compound to participate in HAT reactions.
(2) 2,2’-azino-bis(3-ethylbenzothiazoline-6-sulfonic acid) (ABTS) assay, involving the stable radical-ion ABTS^●+^. This stable radical-ion is regularly used to investigate the ability of a compound to participate in SET reactions;

The results of both assays on the ligand 2b′ and its complexes Ln(2b′)_3_ are shown in Figure 10.

The ligand 2b′ was tested at molarities between 1 × 10^−6^ M and 1 × 10^−4^ M, while the corresponding Ln complexes were tested at three times lower concentrations. As each complex incorporates three ligands, this allows the authors to compare the impact of the coordination center on the reactivity of the ligands in the presence of these model systems. The results are presented as radical-scavenging activity (RSA); higher values mean greater scavenging of the stable free radicals. The results for the ligand 2b′ and its La(III) complex have previously been published [15]. The present article expands with data on the DPPH and ABTS scavenging activity of 2b′ complexes with seven more Ln(III) ions. 

In terms of participation of the ligand and its Ln complexes in HAT with DPPH^●^ (Figure 10a), what can be noted is that the ligand 2b′ is completely inactive, its RSA being statistically zero, within the entire range of tested molarities. Coordinating it with Ln(III) ions causes a mild, concentration-dependent, DPPH-scavenging activity at 3 × 10^−5^ M. Overall, all complexes exhibited RSA between about 3% and 5% at this molarity. Two clear outliers to this trend can be observed: La(2b′)_3_ (RSA = 9.8 ± 0.8%) and Sm(2b′)_3_ (RSA = 10.3 ± 0.9%).

Electron exchange reactions with ABTS^●+^ (Figure 10b) seem to be more prominent, compared to HAT with DPPH^●^ with both 2b′ and its Ln(III) complexes. At 1 × 10^−4^ M, 2b′ behaves as a mild scavenger of ABTS^●+^ (RSA = 16.9 ± 0.7%). In this case, what can be observed is that the effect of Ln coordination on SET reactions with ABTS seems to be suppressive. At 3 × 10^−5^ M, almost all complexes manifest RSA between 6% and 11%. Interestingly, in this case, two clear outliers also stand out: Ce(2b′)_3_ (RSA = 23.4 ± 1.1%) and Ho(2b′)_3_ (RSA = 22.3 ± 3.1%).

## 3. Methods and Materials

### 3.1. Experimental Details

The methodology for the synthesis of Ln(III) complexes has been previously reported by us [15]. Infrared spectra of the Ln(2b′)_3_ complexes (Ln = La, Ce, Pr, Nd, Sm, Gd, Dy, Ho and Er) were recorded as KBr pellets in the solid-state sample in the 4000–400 cm^−1^ range by a Bruker spectrometer, model FTIR IFS25. Raman spectra of these complexes were recorded in the solid state sample in the 4000–50 cm^−1^ range using a Dilor microspectrometer (model LabRam of Horiba-Jobin-Yvon), which is prepared with a holographic grating of 1800 grooves/mm. The spectra have been recorded in backscattering geometry using a confocal Raman microscope, which is prepared with a 50× objective model OlympusLMPlanFL. Raman signal was detected using a Peltier-cooled CCD camera. The Laser power of 100 mW was utilized in our measurements.

The DPPH and ABTS assays were performed according to well-established protocols [28,29,30,31]. All materials used were pro-analytical grade SIGMA-ALDRICH (Sigma-Aldrich Chemie GmbH, Taufkirchen, Germany). The ligand 2b′ and its Ln(III) complexes were dissolved in bi-distilled water. Thus prepared, the stock solutions were further diluted in order to obtain the required molarities. A 0.6 mM stock solution of DPPH^●^ in ethanol was prepared and further diluted to achieve an absorbance between 0.5 and 0.9 at 520 nm. A solution of the stable radical ABTS^●+^ was prepared prior to experimentation in the following way: ABTS was dissolved at pH = 3.6 in 3 × 10^−2^ mmol acetate buffer, and H_2_O_2_ was therefore added to produce the stable radical-ion with an absorption peak at 660 nm. In both DPPH and ABTS assays, two types of samples were tested on a SHIMADZU UV-1601 double beam spectrophotometer “sample”, which includes the solution of the compound tested at the desired concentration, and “control”, which includes bi-distilled water, instead of the tested compound. The results were presented as RSA and calculated as follows:

$$RSA,\;\%=\frac{A_{sample}}{A_{control}}\times 100$$

where *A_sample_* and *A_control_* are the measured absorbances of the “sample” and “control” measurements at the appropriate wavelengths.

### 3.2. Computational Details

Due to the large size of the Ln(ligand)_3_ complexes studied, the calculations were only carried out by Density Functional methods (DFT) [32], which have provided results in biomolecules in good accordance with those obtained with the sophisticated MP2 method [33]. As a DFT method, the Minnesota functional M06-2X was selected because it appears to be the best choice among other meta-generalized gradient functionals to examine dispersion-bound systems [34,35]. The CAM-B3LYP DFT method was also chosen since it also provides good results for noncovalent weak interactions, as they are expected in our large systems. In addition, these methods also show broad applicability in chemistry [36]. Finally, the DFT method B3LYP was also selected because it is the most used today for many systems, and it leads to excellent results in the theoretical calculation of the IR and Raman spectra, facilitating their assignments [37]. All these methods are implemented in the GAUSSIAN-16 program package [38], which was running under the UNIX version with standard parameters of this package, in the Brigit super computer of the University Complutense at Madrid.

Few basis sets appear available for lanthanide atoms. The small CEP-4g basis set is available for all of them, and therefore, it was mainly used, especially for comparison purposes. For special cases, the LANL2DZ basis set was also chosen for La-complexes because it has provided good results in previous calculations [15].

In all optimization processes, the harmonic wavenumber calculations were included at the same level to confirm the local minima energy of all optimized complexes, which show only positive harmonic vibrations. A positive charge was necessary to be included in Ce and Nd complexes for the optimization, perhaps due to the special characteristics of these atoms and the small basis set available for their study.

## 4. Conclusions

The effect of La, Ce, Pr and Nd lanthanide ions on four Ln(ligand)_3_ complexes, three of them based on the 1,2,3-triazole ring and at three DFT levels of calculation, was analyzed. The main conclusions were as follows:

- The spatial arrangement of the ligands in these complexes depends on the theoretical method, the basis set used and the lanthanide atom. This arrangement is nearly symmetric by CAM-B3LYP and M06-2X methods and the Lanl2dz basis set, while it is deformed by B3LYP and the Cep-4g and Lanl2mb basis sets.
- Relationships have been established between the geometric parameters, atomic charges, HOMO-LUMO energies and other molecular properties. As the size of the ligand increases, the dipole moment is incremented. The reduction in the Ln atomic charge leads to a lengthening of the La-O12 and La-O13 bonds. As the ligand becomes larger and more complex, the Ln charge is increased, and this effect is more pronounced in Ln ions with large positive charge.
- Increasing the ligand size significantly reduces the HOMO energy. The high energy gap Eg calculated in A- and B-complexes reveal that they have low polarizability, with low chemical reactivity and high kinetic stability, while C- and Ln(2b′)_3_ complexes with very low Eg values indicate that they have large chemical reactivity and small excitation energies to the manifold of excited states.
- Due to the lowest values of η and *S* in C- and Ln(2b′)_3_ complexes, they can be described as soft with a small gap and with an electron density that can change easily.
- Due to the spatial arrangement of the ligands in the Ln(2b′)_3_ complexes, hydrated water molecules appear in their structure by the appearance of a broad ν(O-H) stretching band corresponding to water molecules in the experimental IR spectrum to the solid-state sample. These hydrated water molecules are H-bonded to the nitrogen atoms and to a large negative charge around three carboxylate groups.
- The experimental IR and Raman spectra of Ln(2b′)_3_ complexes where Ln = La, Ce, Pr, Nd, Sm, Gd, Dy, Ho and Er ions were recorded and compared.
- The arrangement of the hydrated water molecules little changes in the Ln(2b′)_3_ complexes with the lanthanide ion according to similar wave number of the experimental ν(O-H) stretching IR band position, but its amount especially increases in the Ho and Er complexes with a broadening of the very strong band at ca. 1578 cm^−1^.
- Coordination of 2b′ with Ln(III) ions seems to improve HAT with DPPH^●^ the complexes exhibit a mild activity compared to the ligand, which is inactive in this model system. This increase in HAT activity is most pronounced in the lanthanum and samarium complexes.
- In the ABTS model system, contrary to DPPH, coordination of 2b′ with Ln(III) ions seems to generally suppress SET activity—the complexes generally scavenge ABTS^●+^ to a lesser extent, compared to the ligand 2b′ at three times higher concentration. The cerium and holmium complexes seem to be an exception to this trend, as their SET activity is higher than that of 2b′ at three times the concentration.
- Similarities in the IR and Raman spectra seem to be reflected as similarities in the activities of the Ln(III) complexes in the presence of the tested in vitro model systems.

Since the crystal structure of these complexes cannot be determined, the theoretical approaches used here and the relationships established may be useful to synthesize new complexes with better chemical properties.

## Figures and Tables

**Table 1:** Several selected bond lengths in Å calculated at three DFT levels in the A-, B-, C- and Ln-(2b′)_3_ complexes with Ln = La, Ce, Pr and Nd.

Bond Length	Ln=	B3LYP				CAM-B3LYP				M06-2X			
		A	B	C	Ln(2b′)_3_	A	B	C	Ln(2b′)_3_	A	B	C	Ln(2b′)_3_
Ln-O12	La	2.473	2.478	2.478	2.257	2.453	2.463	2.465	2.441	2.468	2.466	2.472	2.472
	Ce	2.287	2.29	2.288	2.304	2.256	2.26	2.257	2.264	2.248	2.261	2.253	2.26
	Pr	2.376	2.376	2.375	2.386	2.373	2.362	2.353	2.366	2.354	2.363	2.36	2.367
	Nd	2.28	2.277	2.313	2.35	2.248	2.291	2.226	2.265	2.285	2.295	2.268	2.274
Ln-O13	La	2.465	2.449	2.448	3.646	2.46	2.445	2.439	2.427	2.432	2.424	2.418	2.417
	Ce	2.286	2.282	2.281	2.275	2.255	2.251	2.251	2.231	2.253	2.24	2.245	2.224
	Pr	2.363	2.372	2.369	2.346	2.346	2.351	2.363	2.348	2.357	2.354	2.365	2.346
	Nd	2.27	2.268	2.285	2.324	2.217	2.183	2.236	2.187	2.181	2.165	2.192	2.162
C11-O12	La	1.421	1.419	1.418	1.418	1.411	1.407	1.406	1.407	1.395	1.393	1.392	1.39
	Ce	1.434	1.434	1.436	1.437	1.423	1.422	1.423	1.425	1.413	1.41	1.413	1.415
	Pr	1.42	1.42	1.421	1.421	1.409	1.408	1.409	1.409	1.399	1.395	1.398	1.396
	Nd	1.432	1.435	1.431	1.43	1.419	1.408	1.424	1.418	1.397	1.395	1.404	1.405
C9-C11	La	1.644	1.594	1.593	1.579	1.635	1.589	1.589	1.574	1.627	1.584	1.584	1.581
	Ce	1.657	1.587	1.583	1.576	1.648	1.581	1.577	1.563	1.638	1.575	1.571	1.556
	Pr	1.667	1.619	1.618	1.612	1.656	1.611	1.611	1.606	1.647	1.605	1.606	1.598
	Nd	1.659	1.588	1.592	1.599	1.65	1.581	1.578	1.562	1.639	1.575	1.572	1.557

**Table 2:** Several selected APT charges calculated at three DFT levels in the A-, B-, C- and Ln-(2b′)_3_ complexes with Ln = La, Ce, Pr and Nd. The dipole moment values (μ, Debye) were also included to establish relationships.

Atom	Ln=	B3LYP				CAM-B3LYP				M06-2X			
		A	B	C	Ln(2b′)_3_	A	B	C	Ln(2b′)_3_	A	B	C	Ln(2b′)_3_
Ln	La	2.253	2.584	2.947	2.884	2.296	2.593	2.885	2.816	2.344	2.637	2.926	2.841
	Ce	2.366	2.982	3.657	2.844	2.814	3.464	4.147	4.457	2.752	3.436	4.117	4.494
	Pr	2.301	2.709	3.137	3.276	2.339	2.717	3.043	3.16	2.408	2.764	3.117	3.235
	Nd	2.333	2.342	0.851	0.526	2.372	2.917	3.615	3.874	2.583	3.19	3.88	4.165
O12	La	−0.761	−0.798	−0.932	−1.014	−0.811	−0.832	−0.945	−1.002	−0.808	−0.856	−0.962	−0.948
	Ce	−0.733	−0.921	−1.205	−0.909	−0.819	−0.995	−1.241	−1.323	−0.794	−0.989	−1.260	−1.363
	Pr	−0.743	−0.822	−0.979	−1.005	−0.772	−0.854	−0.988	−1.005	−0.825	−0.878	−1.052	−1.053
	Nd	−0.739	−0.828	−0.356	−0.227	−0.768	−0.858	−1.235	−1.263	−0.697	−0.903	−1.187	−1.287
O13	La	−0.776	−0.837	−0.956	−1.089	−0.800	−0.866	−0.964	−1.065	−0.876	−0.930	−1.039	−1.078
	Ce	−0.747	−0.937	−1.167	−0.925	−0.823	−1.011	−1.224	−1.380	−0.797	−0.992	−1.201	−1.413
	Pr	−0.762	−0.837	−0.972	−1.059	−0.799	−0.859	−0.952	−1.030	−0.832	−0.918	−1.014	−1.109
	Nd	−0.788	−0.894	−0.333	−0.161	−0.825	−0.983	−1.196	−1.339	−0.873	−1.060	−1.379	−1.434
C11	La	0.732	1.032	1.261	1.381	0.782	1.002	1.272	1.368	0.863	1.162	1.359	1.258
	Ce	0.83	1.459	1.954	1.458	0.831	1.446	1.871	2.071	0.81	1.441	1.9	2.134
	Pr	0.689	1.001	1.24	1.363	0.73	1.045	1.238	1.353	0.81	1.123	1.348	1.485
	Nd	0.913	1.577	0.529	0.259	0.954	1.522	2.019	2.375	0.884	1.511	2.136	2.208
μ	La	0.069	0.042	0.734	4.377	0.044	0.028	0.861	2.909	0.571	0.463	0.527	8.038
	Ce	0.101	0.117	4.595	11.523	0.098	0.04	5.416	10.916	0.13	0.036	2.571	10.604
	Pr	0.111	0.211	2.394	9.726	0.233	0.17	1.114	4.375	0.159	0.294	1.277	3.802
	Nd	0.143	0.243	1.589	6.885	0.162	0.958	5.268	7.513	0.152	0.3	4.161	6.175

**Table 3:** Several selected molecular properties calculated at three DFT levels in the A-, B-, C- and Ln-(2b′)_3_ complexes with Ln = La, Ce, Pr and Nd. HOMO and LUMO energies in eV, energy gap (Eg) and global softness (S) in eV, and capacity at constant volume (Cv) in cal/mol·K.

Atom	Ln=	B3LYP				CAM-B3LYP				M06-2X			
		A	B	C	Ln(2b′)_3_	A	B	C	Ln(2b′)_3_	A	B	C	Ln(2b′)_3_
HOMO	La	−0.296	−0.316	−0.302	−0.253	−0.357	−0.378	−0.352	−0.300	−0.372	−0.390	−0.349	−0.300
	Ce	−0.490	−0.456	−0.375	−0.342	−0.553	−0.512	−0.423	−0.390	−0.565	−0.513	−0.421	−0.390
	Pr	−0.296	−0.320	−0.302	−0.252	−0.358	−0.381	−0.351	−0.300	−0.371	−0.391	−0.349	−0.300
	Nd	−0.489	−0.455	−0.377	−0.337	−0.554	−0.506	−0.424	−0.384	−0.566	−0.510	−0.419	−0.389
LUMO	La	−0.090	−0.145	−0.159	−0.146	−0.034	−0.093	−0.110	−0.096	−0.052	−0.105	−0.122	−0.121
	Ce	−0.367	−0.361	−0.347	−0.306	−0.290	−0.288	−0.279	−0.239	−0.350	−0.346	−0.337	−0.296
	Pr	−0.122	−0.149	−0.158	−0.140	−0.079	−0.091	−0.108	−0.092	−0.068	−0.103	−0.122	−0.107
	Nd	−0.389	−0.383	−0.362	−0.316	−0.325	−0.329	−0.315	−0.277	−0.341	−0.338	−0.331	−0.289
Eg	La	0.206	0.171	0.143	0.107	0.323	0.285	0.242	0.204	0.32	0.285	0.227	0.179
	Ce	0.123	0.095	0.028	0.107	0.263	0.224	0.144	0.151	0.214	0.167	0.083	0.093
	Pr	0.174	0.171	0.144	0.112	0.279	0.29	0.243	0.208	0.303	0.288	0.227	0.193
	Nd	0.1	0.072	0.015	0.021	0.229	0.177	0.109	0.107	0.225	0.172	0.088	0.1
S	La	0.051	0.043	0.036	0.027	0.081	0.071	0.06	0.051	0.08	0.071	0.057	0.045
	Ce	0.1	0.024	0.007	0.009	0.066	0.056	0.036	0.038	0.054	0.042	0.021	0.023
	Pr	0.043	0.043	0.036	0.028	0.07	0.072	0.061	0.052	0.076	0.072	0.057	0.048
	Nd	0.025	0.018	0.004	0.005	0.057	0.044	0.027	0.027	0.056	0.043	0.022	0.025
Cv	La	56.2	89.2	167.5	229.9	51.6	87	163.5	224.8	55.2	85.5	161.7	222.1
	Ce	58.5	89.3	168.1	231.5	51.7	86.8	163.8	231	53.6	83.8	162.4	225.9
	Pr	52.4	89.6	167.9	231	57.6	87.3	163.8	225.9	55.4	85.9	162.2	221.9
	Nd	58.5	89.3	168.4	231.9	53.7	86.7	161.8	224.2	53.5	83.7	160.3	223.8

## Data Availability

The data presented in this study are available in this article and supplementary material.

## Supplementary Materials

The following supporting information can be downloaded at: https://www.mdpi.com/article/10.3390/ijms25147964/s1.
