# Supplementary material for: Effect of Lanthanide Ions and Triazole Ligands on the Molecular Properties, Spectroscopy and Pharmacological Activity
Source: Int J Mol Sci. 2024 Jul 21;25(14):7964. doi: 10.3390/ijms25147964 (PMC11276792; doi:10.3390/ijms25147964)
Supplement: Supplementary file 1 [file ijms-25-07964-s001.zip › ijms-3097698-supplementary.pdf]

Effect of lanthanide ions and triazole ligands on the molecular properties, spectroscopy and pharmacological activity

Mauricio Alcolea Palafox [1]*, Nataliya P. Belskaya [2], Lozan T. Todorov[3]*, Nadya G. Hristova-Avakoumova[4] and Irena P. Kostova [3]

[1] Departamento de Química Física, Facultad de Ciencias Químicas, Universidad Complutense, Madrid-28040, Spain (alcolea@ucm.es)
[2] Department of Technology for Organic Synthesis, Ural Federal University, 19 Mira Str., Yekaterinburg 620012, Russia; n.p.belskaya@urfu.ru
[3] Department of Chemistry, Faculty of Pharmacy, Medical University – Sofia, 2 Dunav Str., Sofia, Bulgaria; ltodorov@pharmfac.mu-sofia.bg, irenakostova@yahoo.com
[4] Department of Medical Physics and Biophysics, Faculty of Medicine, Medical University of Sofia, 2 Zdrave Str., 1431 Sofia, Bulgaria

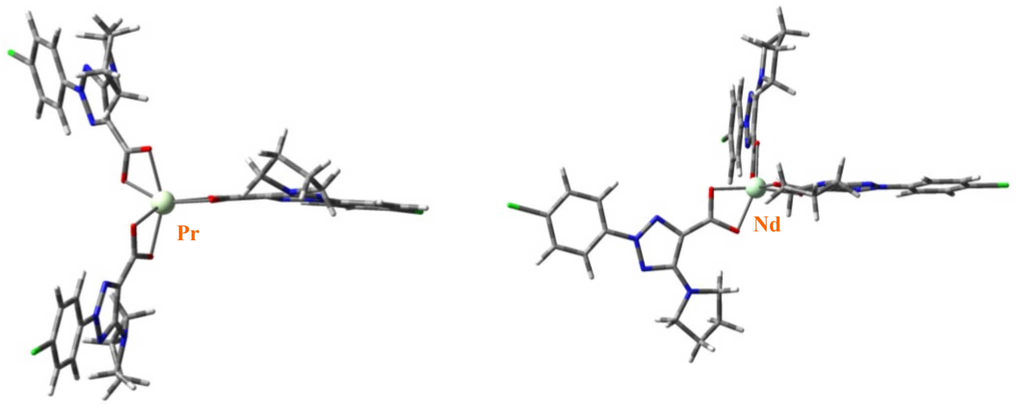

Figure S1. Optimized structure of the Pr(2b′)$_3$ and Nd(2b′)$_3$ complexes with different arrangement of the ligands, obtained at the CAM-B3LYP/Cep-4g level.

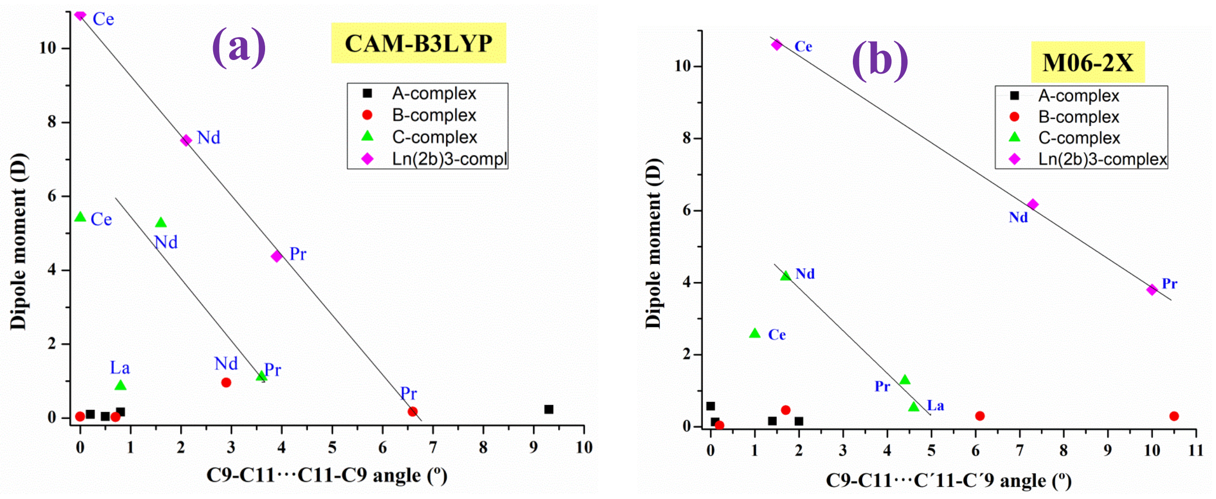

Figure S2. Relationships established between the dipole moment and the torsional angle between two ligands, using the CAM-B3LYP and M06-2X methods.

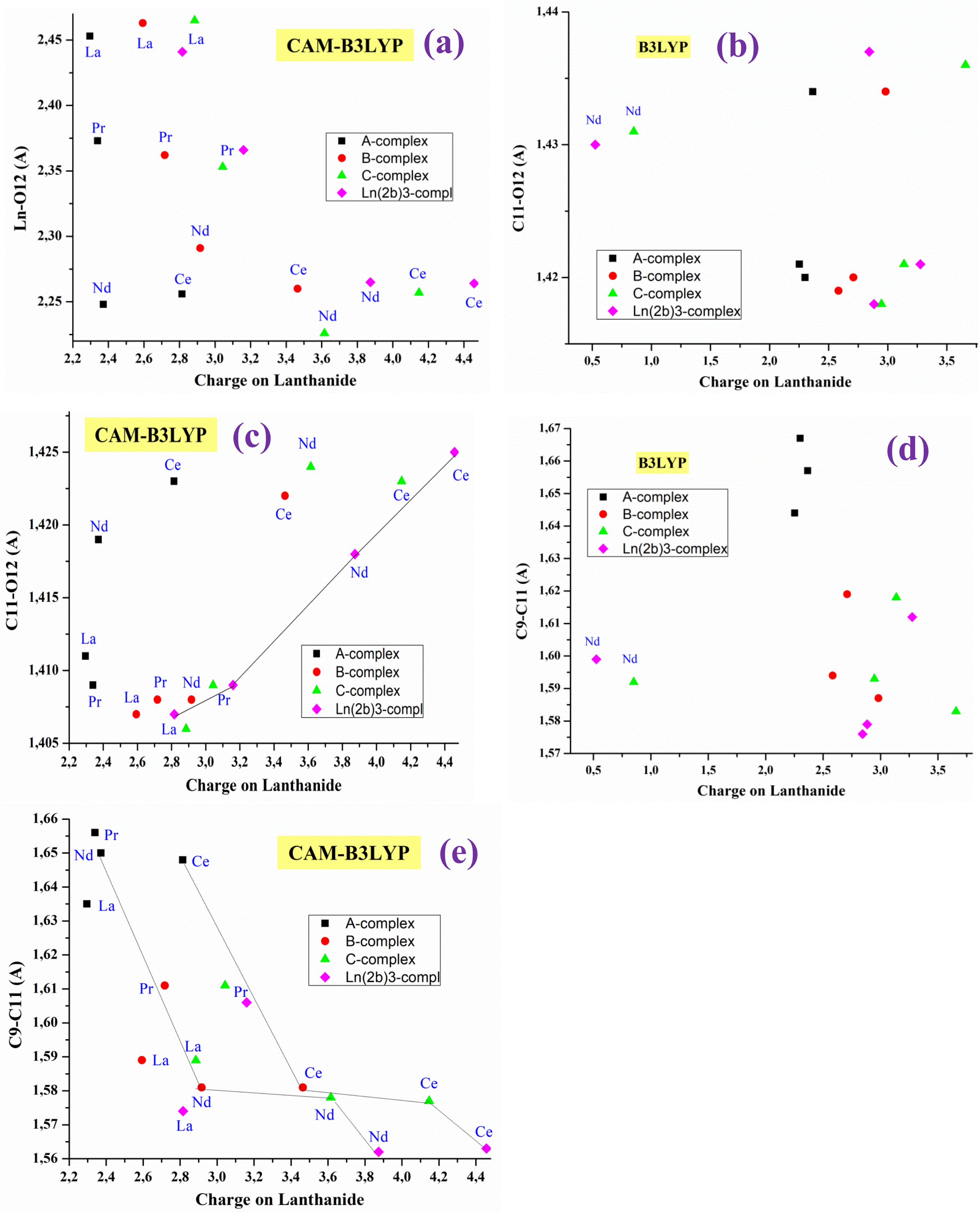

Figure S3. Relationships established between the charge on the lanthanide atom with the Ln-O12, C11-O12, C9-C11 bond lengths, using the B3LYP and CAM-B3LYP methods.

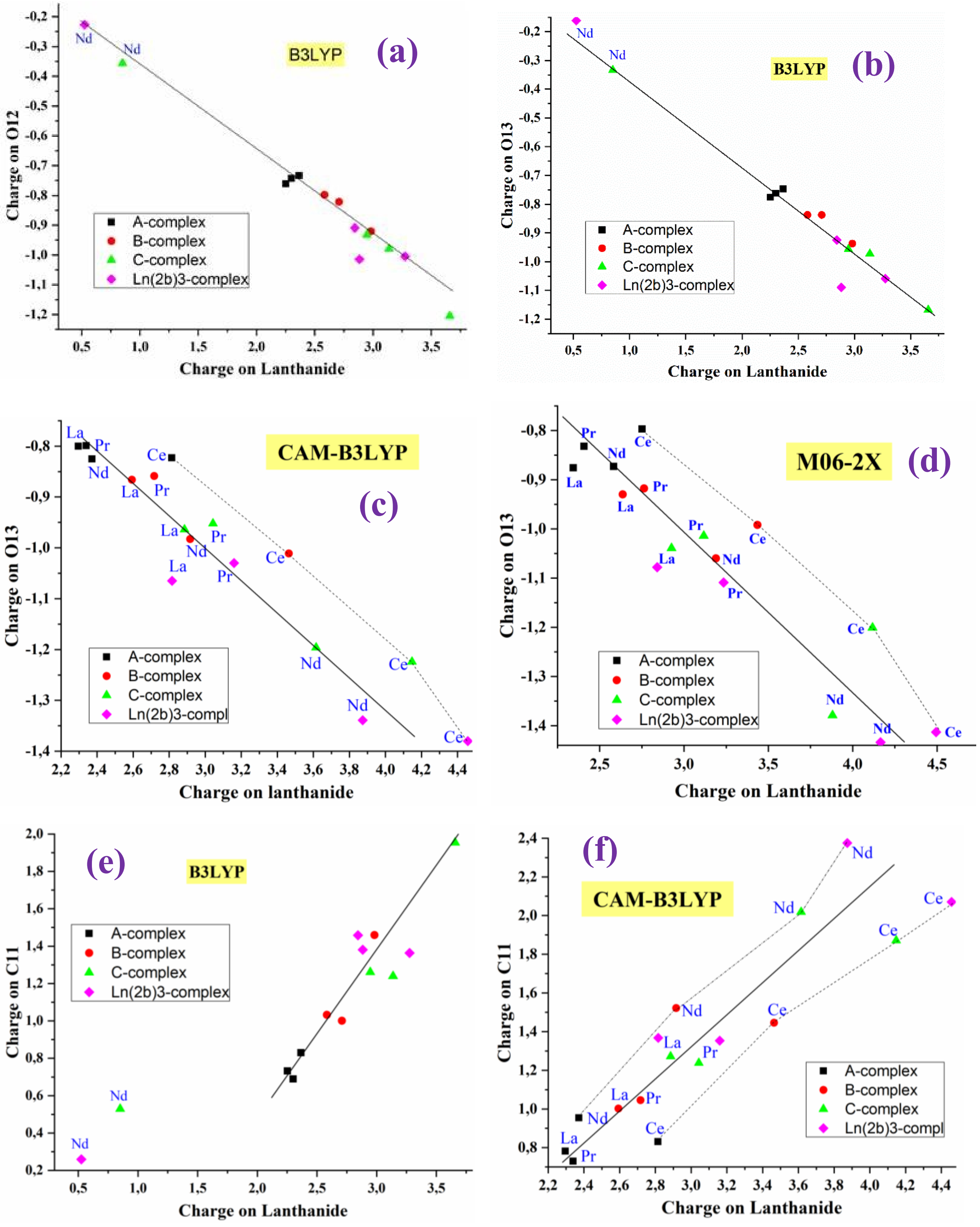

Figure S4. Relationships established between the charge on the lanthanide atom with the charge on O12, O13 and C11 atom with the B3LYP, CAM-B3LYP and M06-2X methods.

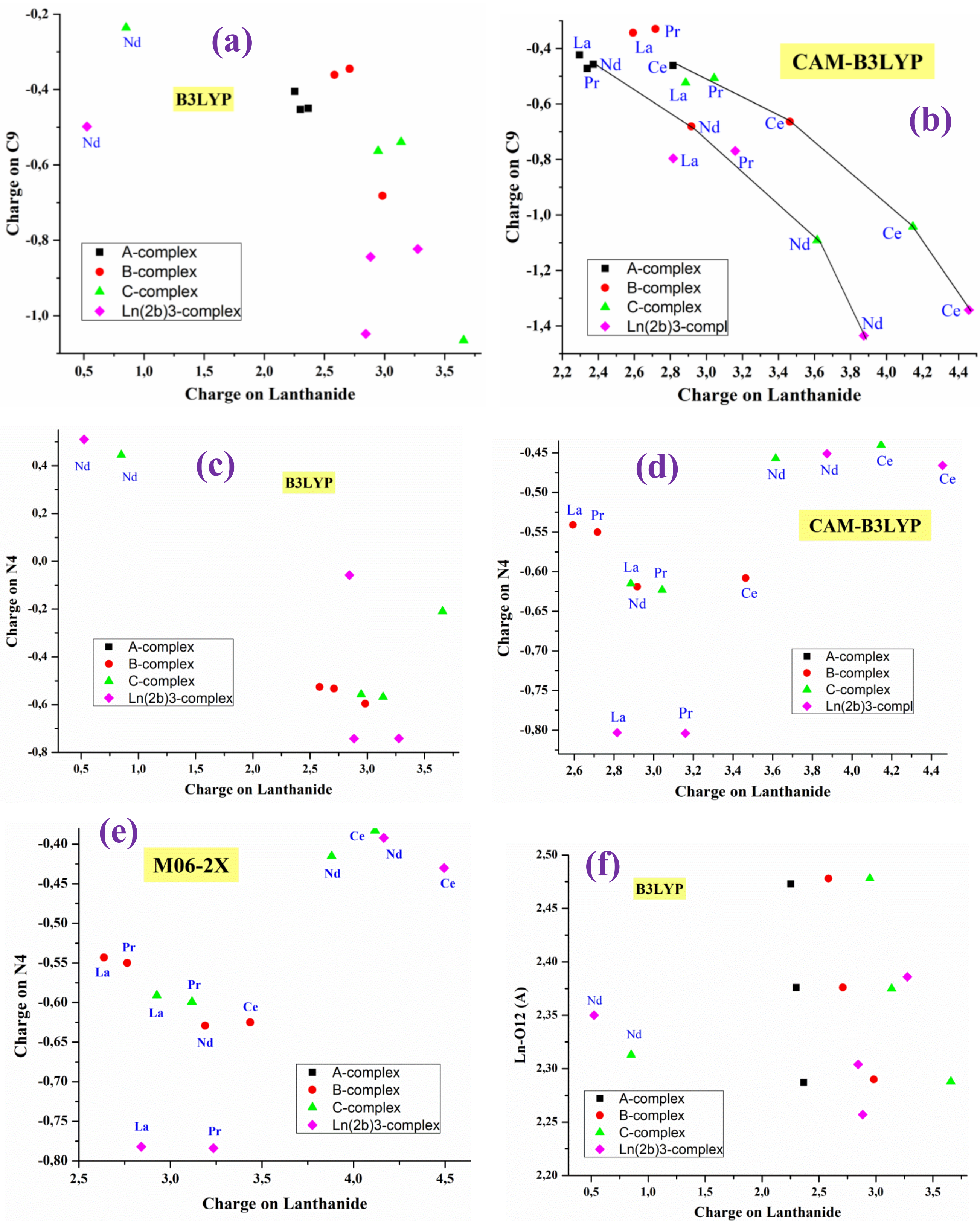

Figure S5. Trying to establish relationships between the charge on the lanthanide atom with the charge on C9 and N4 atoms and with the Ln-O12 bond length, using the B3LYP, CAM-B3LYP and M06-2X methods.

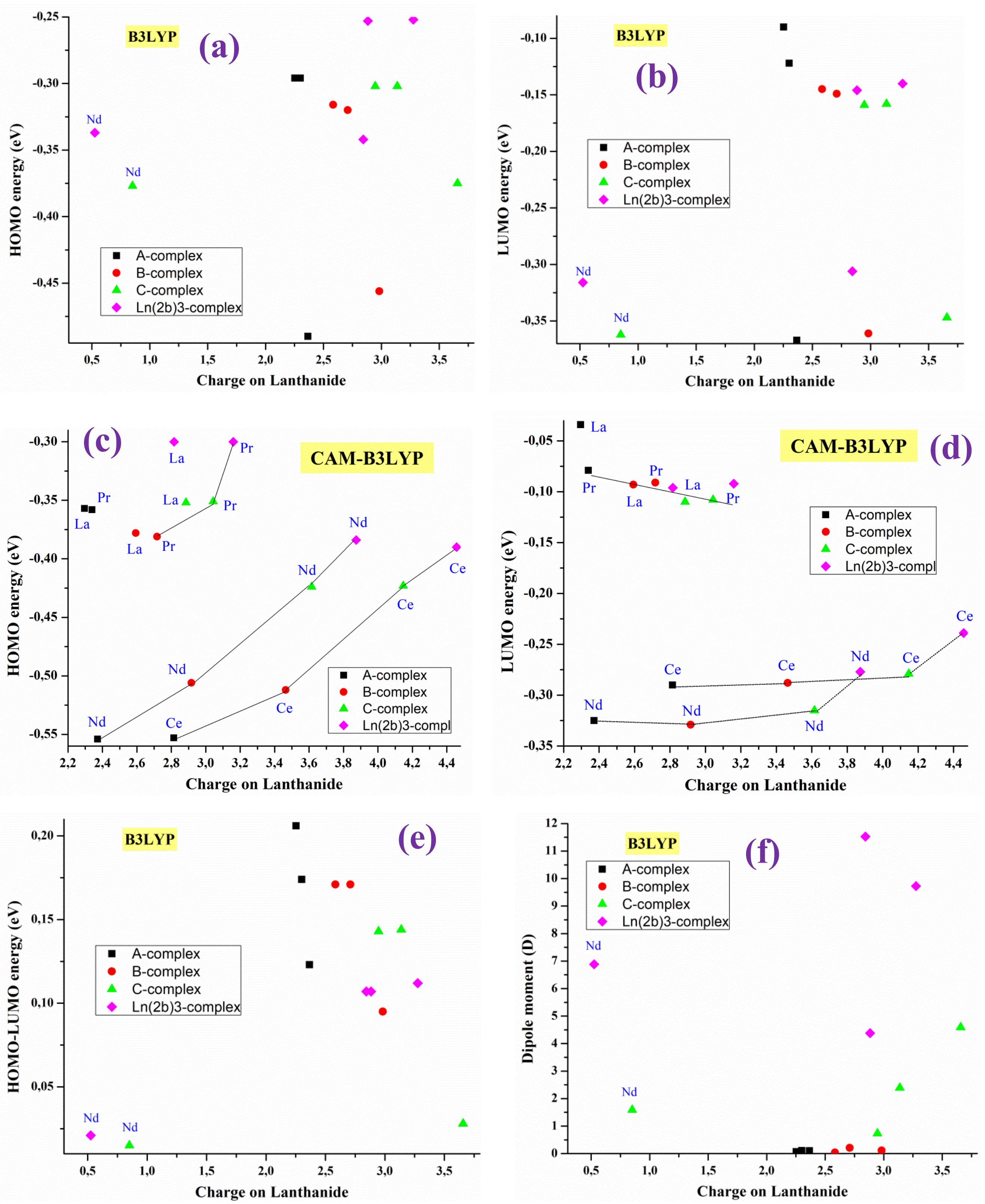

Figure S6. Possible relationships established between the charge on the lanthanide atom with the HOMO and LUMO energies, and with the dipole moment, using the B3LYP and CAM-B3LYP methods.

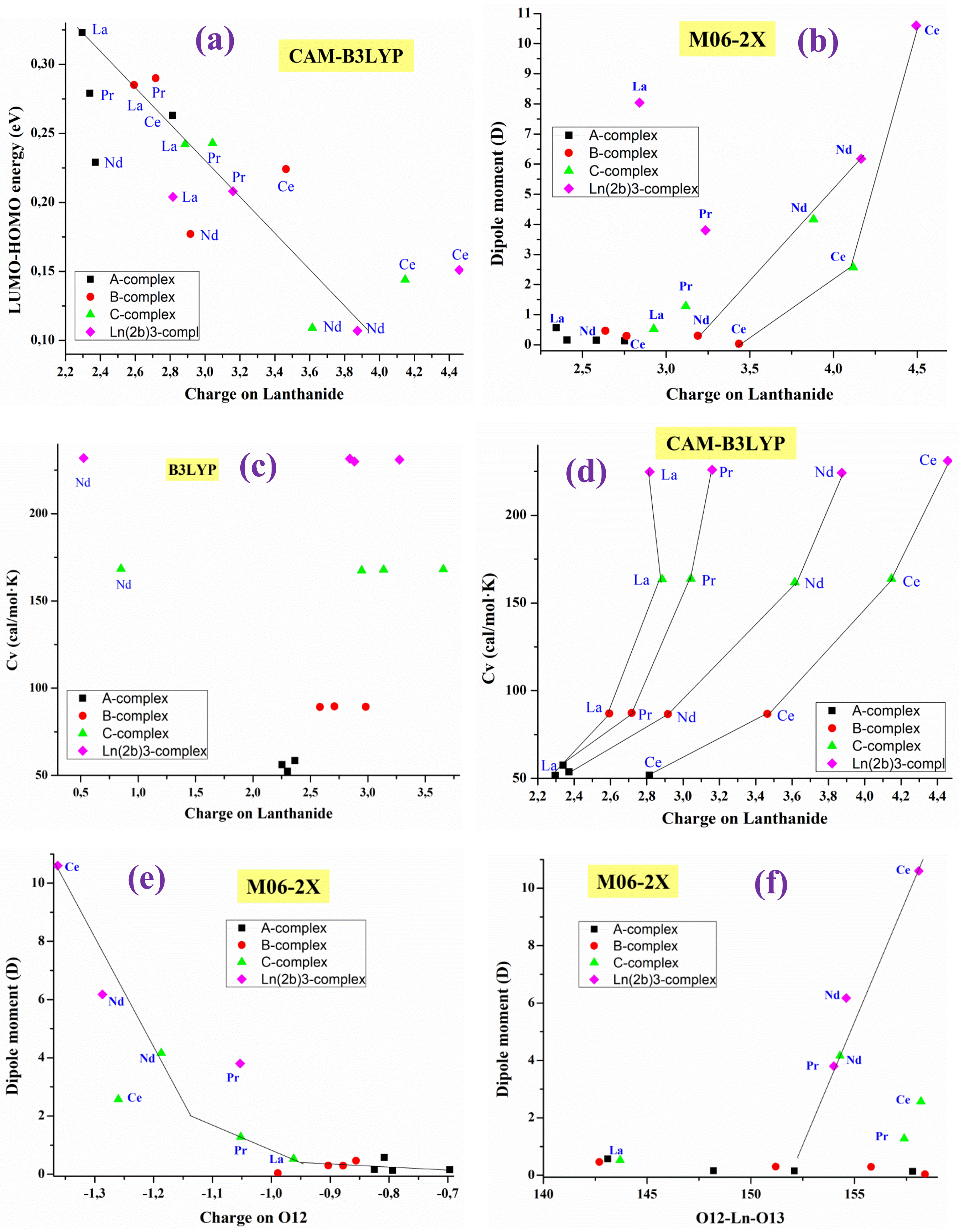

Figure S7. Relationships established between the charge on the lanthanide atom with the LUMO-HOMO energies, dipole moment and Cv, as well as relationships between the dipole moment and the charge on O12, and the O-Ln-O angle, using the B3LYP, CAM-B3LYP and M06-2X methods.

Table S1. Optimized geometrical parameters, bond lengths in Å, and bond angles and torsional angles in degrees, calculated with three DFT methods and the Cep-4g basis set in the A-, B-, C- and La(2b′)$_3$ complexes under study.

**La**	**A-**			**B-**			**C-**			**La(2b′)$_3$**		
Parameters	B3LYP	CAM-B3LYP	M06-2X/	B3LYP/	CAM-B3LYP	M06-2X	B3LYP	CAM-B3LYP	M06-2X/	B3LYP	CAM-B3LYP	M06-2X/
r(C_9-C_{11})	1.644	1.635	1.627	1.594	1.589	1.584	1.593	1.589	1.584	1.579	1.574	1.581
r(C=O_{12})	1.421	1.411	1.395	1.419	1.407	1.393	1.418	1.406	1.392	1.418	1.407	1.390
r(C=O_{13})	1.423	1.410	1.403	1.420	1.407	1.398	1.421	1.408	1.399	1.428	1.413	1.401
r(La-O_{12})	2.473	2.453	2.468	2.478	2.463	2.466	2.478	2.465	2.472	2.257	2.441	2.472
r(La-O_{13})	2.465	2.460	2.432	2.449	2.445	2.424	2.448	2.439	2.418	3.646	2.427	2.417
∠(C_9-C_{11}=O_{12})	121.5	121.7	121.9	118.9	119.1	119.1	119.1	119.3	119.3	121.3	120.8	120.5
∠(O=C=O)	117.1	116.6	116.6	119.3	118.6	118.8	119.4	118.9	119.0	117.9	117.7	117.5
∠(C=O_{12}-La)	91.9	92.6	92.0	90.0	90.7	90.4	89.9	90.4	90.0	87.9	88.4	87.5
∠(C=O_{13}-La)	92.2	92.3	93.3	91.1	91.5	92.0	91.1	91.4	92.1	89.0	88.8	89.4
∠(O_{12}-La-O'_{13})	139.6	141.5	143.1	138.2	141,8	142.7	137.3	141.3	143.7	95.2	142.7	120.4
∠(O_{13}-La-O'_{12})	116.9	114.1	108.8	118.6	114.2	111.2	119.9	115.2	111.2	119.2	100.1	135.2
∠(C_{11}-O_{12}···O'_{12}-C'_{11})	25.5	31.2	40.6	24.1	34.2	43.0	21.4	32.1	47.3	-81.9	90.0	4.7
∠(C_9-C_{11}···C'_{11}-C'_9)	-0.7	0.5	0.0	-0.9	-0.7	-1.7	-0.9	-0.8	-4.6	-52.7	-44.6	-15.3
∠(C'_9-C'_{11}···C''_{11}-C''_9)	0.6	0.1	1.2	5.2	5.6	7.3	4.2	4.6	7.0	-21.1	26.4	-5.1
∠(C_{11}···La··· C'_{11})	120.3	120.0	117.6	120.7	120.5	120.3	120.9	120.8	121.1	145.6	112.7	118.1
∠(C'_{11}···La··· C''_{11})	119.7	119.9	119.4	119.9	120.1	117.0	119.7	120.0	116.2	91.1	88.3	93.5
**Ce**.	**A-**			**B-**			**C-**			**Ce(2b′)$_3$**		
Parameters	B3LYP	CAM-B3LYP	M06-2X/	B3LYP/	CAM-B3LYP	M06-2X	B3LYP	CAM-B3LYP	M06-2X/	B3LYP	CAM-B3LYP	M06-2X/
r(C_9-C_{11})	1.657	1.648	1.638	1.587	1.581	1.575	1.583	1.577	1.571	1.576	1.563	1.556
r(C=O_{12})	1.434	1.423	1.412	1.434	1.422	1.410	1.436	1.423	1.413	1.437	1.425	1.415
r(C=O_{13})	1.434	1.423	1.412	1.435	1.423	1.414	1.436	1.423	1.413	1.442	1.433	1.425
r(Ce-O_{12})	2.287	2.256	2.248	2.290	2.260	2.261	2.288	2.257	2.253	2.304	2.264	2.260
r(Ce-O_{13})	2.286	2.255	2.253	2.282	2.251	2.240	2.281	2.251	2.245	2.275	2.231	2.224
∠(C_9-C_{11}=O_{12})	123.7	123.9	123.4	122.2	122.4	122.0	122.1	122.4	121.7	125.9	126.6	125.5
∠(O=C=O)	112.5	112.1	113.2	114.2	113.8	114.8	114.2	113.7	114.8	113.7	112.7	113.8

∠(C=O$_{12}$-Ce)	92.3	92.3	91.9	90.9	91.0	90.3	90,9	91.1	90.5	90.9	91.1	90.4
∠(C=O$_{13}$-Ce)	92.3	92.4	91.7	91.2	91.4	91.0	91.2	91.3	90.8	91.9	92.2	91.6
∠(O$_{12}$-Ce-O′$_{13}$)	153.9	155.3	157.8	154.3	155.5	158.4	153.8	155.3	158.2	152.4	154.8	158.1
∠(O$_{13}$-Ce-O′$_{12}$)	102.2	100.8	97.4	102.7	101.2	97.5	103.1	101.3	97.2	104.5	101.6	99.2
∠(C$_{11}$-O$_{12}$···O′$_{12}$-C′$_{11}$)	57.6	60.6	66.2	56.9	60.5	67.3	55.9	59.8	68.3	56.4	61.7	61.8
∠(C$_9$-C$_{11}$···C′$_{11}$-C′$_9$)	-0.1	-0.2	0.1	0.2	0.0	0.2	0.0	0.0	1.0	-0.2	0.0	-1.5
∠(C′$_9$-C′$_{11}$···C″$_{11}$-C″$_9$)	0.1	0.3	-0.4	2.1	2.6	1.8	0.0	0.1	2.7	-0.1	-0.1	-18.0
∠(C$_{11}$···Ce··· C′$_{11}$)	120.1	120.2	119.9	120.2	120.2	118.9	119,9	120.1	119.7	120.0	119.8	120.9
∠(C′$_{11}$···Ce··· C″$_{11}$)	120.0	119.6	120.1	119.0	119.0	120.9	120.1	120.0	120.1	119.8	119.9	120.3
**Pr**		**A-**			**B-**			**C-**			**Pr(2b′)$_3$**	
r(C$_9$-C$_{11}$)	1.667	1.656	1.647	1.619	1.611	1.605	1.618	1.611	1.606	1.612	1.606	1.598
r(C=O$_{12}$)	1.420	1.409	1.399	1.420	1.408	1.395	1.421	1.409	1.398	1.421	1.409	1.396
r(C=O$_{13}$)	1.422	1.411	1.399	1.420	1.407	1.397	1.419	1.405	1.395	1.426	1.411	1.401
r(Pr-O$_{12}$)	2.376	2.373	2.354	2.376	2.362	2.363	2.375	2.353	2.360	2.386	2.366	2.367
r(Pr-O$_{13}$)	2.363	2.346	2.357	2.372	2.351	2.354	2.369	2.363	2.365	2.346	2.348	2.346
∠(C$_9$-C$_{11}$=O$_{12}$)	121.5	122.1	121.5	119.0	119.4	119.1	119.2	119.1	119.1	123.6	122.6	122.3
∠(O=C=O)	117.4	116.9	116.9	119.2	118.7	118.6	119.2	118.8	118.7	117.7	117.7	117.7
∠(C=O$_{12}$-Pr)	90.3	90.3	91.2	89.3	89.4	89.7	89.1	89.8	90.1	89.4	90.0	90.2
∠(C=O$_{13}$-Pr)	90.7	91.4	91.0	89.4	89.9	90.1	89.4	89.5	90.0	90.9	90.6	90.9
∠(O$_{12}$-Pr-O′$_{13}$)	152.5	153.8	148.2	153.7	153.7	155.8	153.4	152.7	157.4	154.6	152.1	154.0
∠(O$_{13}$-Pr-O′$_{12}$)	103.8	106.6	101.2	101.4	99.4	105.6	103.6	99.8	108.9	104.4	102.3	107.0
∠(C$_{11}$-O$_{12}$···O′$_{12}$-C′$_{11}$)	56.3	52.1	55.5	62.4	57.6	69.8	60.2	61.9	53.8	58.5	57.1	63.7
∠(C$_9$-C$_{11}$···C′$_{11}$-C′$_9$)	-1.5	-9.3	1.4	1.4	6.6	-10.5	-5.1	3.6	-4.4	-4.2	3.9	-10.0
∠(C′$_9$-C′$_{11}$···C″$_{11}$-C″$_9$)	-0.5	-2.8	0.9	1.1	0.2	6.8	8.1	2.9	8.3	-8.4	-11.0	-3.7
∠(C$_{11}$···Pr··· C′$_{11}$)	120.6	124.6	116.0	119.6	117.6	125.5	121.5	117.5	127.3	123.0	118.9	126.5
∠(C′$_{11}$···Pr··· C″$_{11}$)	119.6	120.8	118.5	116.9	121.3	113.4	116.1	122.1	114.3	120.6	120.2	110.8
**Nd**		**A-**			**B-**			**C-**			**Nd(2b′)$_3$**	
r(C$_9$-C$_{11}$)	1.659	1.650	1.639	1.588	1.581	1.575	1.592	1.578*	1.572	1.599	1.562	1.557
r(C=O$_{12}$)	1.432	1.419	1.397	1.435	1.408	1.395	1.431	1.424	1.404	1.430	1.418	1.405
r(C=O$_{13}$)	1.437	1.429	1.429	1.437	1.443	1.432	1.436	1.423	1.425	1.428	1.447	1.437
r(Nd-O$_{12}$)	2.280	2.248	2.285	2.277	2.291	2.295	2.313	2.226	2.268	2.350	2.265	2.274
r(Nd-O$_{13}$)	2.270	2.217	2.181	2.268	2.183	2.165	2.285	2.236	2.192	2.324	2.187	2.162

$\angle(C_9\text{-}C_{11}{=}O_{12})$	123.8	124.3	124.8	122.3	123.6	123.7	122.4	122.0	122.8	124.4	127.0	128.0
$\angle(O{=}C{=}O)$	112.8	112.5	112.7	114.3	114.2	114.1	115.1	114.0	114.2	116.1	113.0	112.9
$\angle(C{=}O_{12}\text{-}Nd)$	91.8	91.2	90.2	90.6	88.9	88.6	90.2	90.7	89.5	90.2	89.8	89.4
$\angle(C{=}O_{13}\text{-}Nd)$	92.0	92.2	93.6	90.9	92.3	93.0	91.2	90.5	92.0	91.3	92.2	93.1
$\angle(O_{12}\text{-}Nd\text{-}O'_{13})$	155.6	157.8	152.1	157.5	155.2	151.2	156.9	159.5	154.3	151.8	156.7	154.6
$\angle(O_{13}\text{-}Nd\text{-}O'_{12})$	97.9	95.6	93.2	98.9	94.1	94.2	102.0	97.1	93.5	106.1	96.5	97.1
$\angle(C_{11}\text{-}O_{12}\cdots O'_{12}\text{-}C'_{11})$	63.4	68.5	60.3	64.8	61.2	59.9	61.4	68.2	62.1	55.2	63.9	65.8
$\angle(C_9\text{-}C_{11}\cdots C'_{11}\text{-}C'_9)$	-0.5	0.8	-2.0	1.1	-2.9	-6.1	-4.3	1.6	-1.7	-4.2	2.1	-7.3
$\angle(C'_9\text{-}C'_{11}\cdots C''_{11}\text{-}C''_9)$	0.2	-4.1	-7.4	1.0	-6.7	1.5	-1.3	-0.9	-2.4	-2.2	-12.0	-12.5
$\angle(C_{11}\cdots Nd\cdots C'_{11})$	118.5	118.4	113.7	120.2	115.8	113.3	122.6	120.4	115.1	121.7	117.6	117.5
$\angle(C'_{11}\cdots Nd\cdots C''_{11})$	120.4	118.5	113.6	117.4	115.9	113.8	117.4	120.4	112.3	118.5	115.2	116.7

Table S2. Calculated APT (Atomic Polar Tensor) charges in the optimized geometrical parameters with three DFT methods and the Cep-4g basis set in the A-, B-, C- and La(2b′)$_3$ complexes under study.

atom	A-			B-			C-			La(2b′)$_3$		
	B3LYP	CAM-B3LYP	M06-2X	B3LYP/	CAM-B3LYP	M06-2X /	B3LYP	CAM-B3LYP	M06-2X	B3LYP	CAM-B3LYP	M06-2X
**La**	2.253	2.296	2.344	2.584	2.593	2.637	2.947	2.885	2.926	2.884	2.816	2.841
N_4	-	-	-	-0.526	-0.541	-0.543	-0.557	-0.615	-0.591	-0.742	-0.803	-0.782
N_7	-	-	-	-0.039	-0.041	-0.050	0.004	0.017	0.011	-0.066	-0.044	-0.039
C_8	-	-	-	-0.180	-0.192	-0.190	-0.282	-0.298	-0.293	0.373	0.394	0.418
C_9	-0.405	-0.423	-0.484	-0.361	-0.343	-0.366	-0.563	-0.523	-0.549	-0.844	-0.796	-0.625
N_{10}	-	-	-	0.115	0.117	0.114	0.272	0.275	0.273	0.401	0.417	0.358
C_{11}	0.732	0.782	0.863	1.032	1.002	1.162	1.261	1.272	1.359	1.381	1.368	1.258
O_{12}	-0.761	-0.811	-0.808	-0.798	-0.832	-0.856	-0.932	-0.945	-0.962	-1.014	-1.002	-0.948
O_{13}	-0.776	-0.800	-0.876	-0.837	-0.866	-0.930	-0.956	-0.964	-1.039	-1.089	-1.065	-1.078
atom	**A-**			**B-**			**C-**			**Ce(2b′)$_3$**		
	B3LYP	CAM-B3LYP	M06-2X/	B3LYP/	CAM-B3LYP	M06-2X	B3LYP	CAM-B3LYP	M06-2X	B3LYP	CAM-B3LYP	M06-2X
**Ce**	2.366	2.814	2,752	2.982	3.464	3.436	3.657	4.147	4.117	2.844	4.457	4.494
N_4	-	-	-	-0.596	-0.608	-0.625	-0.210	-0.440	-0.383	-0.058	-0.466	-0.430
N_7	-	-	-	-0.013	-0.018	-0.031	-0.111	-0.076	-0.105	-0.099	-0.124	-0.133
C_8	-	-	-	-0.071	-0.090	-0.079	0.021	-0.093	-0.072	0.444	0.596	0.610
C_9	-0.450	-0.461	-0.512	-0.682	-0.664	-0.695	-1.065	-1.042	-1.100	-1.048	-1.342	-1.392
N_{10}	-	-	--	0.253	0.262	0.276	0.335	0.443	0.472	0.105	0.411	0.426
C_{11}	0.830	0.831	0.810	1.459	1.446	1.441	1.954	1.871	1.900	1.458	2.071	2.134
O_{12}	-0.733	-0.819	-0.794	-0.921	-0.995	-0.989	-1.205	-1.241	-1.260	-0.909	-1.323	-1.363
O_{13}	-0.747	-0.823	-0.797	-0.937	-1.011	-0.992	-1.167	-1.224	-1.201	-0.925	-1.380	-1.413
	**A-**			**B-**			**C-**			**Pr(2b)$_3$**		

**Pr**	2.301	2.339	2.408	2.709	2.717	2.764	3.137	3.043	3.117	3.276	3.160	3.235
N_4	-	-	-	-0.533	-0.550	-0.550	-0.568	-0.623	-0.599	-0.741	-0.804	-0.784
N_7	-	-	-	-0.037	-0.039	-0.047	0.006	0.019	0.010	-0.072	-0.045	-0.059
C_8	-	-	-	-0.200	-0.209	-0.207	-0.305	-0.321	-0.312	0.414	0.416	0.428
C_9	-0.453	-0.472	-0.533	-0.345	-0.330	-0.355	-0.539	-0.507	-0.539	-0.823	-0.769	-0.830
N_{10}	-	-	-	0.116	0.119	0.119	0.273	0.279	0.279	0.377	0.392	0.414
C_{11}	0.689	0.730	0.810	1.001	1.045	1.123	1.240	1.238	1.348	1.363	1.353	1.485
O_{12}	-0.743	-0.772	-0.825	-0.822	-0.854	-0.878	-0.979	-0.988	-1.052	-1.005	-1.005	-1.053
O_{13}	-0.762	-0.799	-0.832	-0.837	-0.859	-0.918	-0.972	-0.952.	-1.014	-1.059	-1.030	-1.109
	**A-**			**B-**			**C-**			**Nd(2b)$_3$**		
**Nd**	2.333	2.372	2.583	2.342	2.917	3.190	0.851	3.615	3.880	0.526	3.874	4.165
N_4	-	-	-	-0.607	-0.619	-0.629	0.445	-0.457	-0.415	0.510	-0.451	-0.392
N_7	-	-	-	-0.019	-0.019	-0.030	-0.023	-0.083	-0.105	0.089	-0.129	-0.145
C_8	-	-	-	-0.065	-0.081	-0.072	0.304	-0.086	-0.050	0.137	0.625	0.639
C_9	-0.438	-0.457	-0.521	-0.708	-0.681	-0.706	-0.236	-1.091	-1.163	-0.498	-1.435	-1.425
N_{10}	-	-	-	0.260	0.269	0.277	-0.361	0.469	0.487	-0.414	0.410	0.409
C_{11}	0.913	0.954	0.884	1.577	1.522	1.511	0.529	2.019	2.136	0.259	2.375	2.208
O_{12}	-0.739	-0.768	-0.697	-0.828	-0.858	-0.903	-0.356	-1.235	-1.187	-0.227	-1.263	-1.287
O_{13}	-0.788	-0.825	-0.873	-0.894	-0.983	-1.060	-0.333	-1.196	-1.379	-0.161	-1.339	-1.434

Table S3. Calculated molecular properties in the optimized geometrical parameters with three DFT methods and the Cep-4g basis set in the A-, B-, C- and La(2b′)$_3$ complexes under study.

Molecular properties **La**	**A-**		**B-**		**C-**		**La(2b)$_3$**		
	CAM-B3LYP	M06-2X	CAM-B3LYP	M06-2X	CAM-B3LYP	M06-2X	B3LYP	CAM-B3LYP	M06-2X
Rotational constants: A	0.513	0.527	0.124	0.127	0.024	0.025	0.035	0.036	0.033
(GHz) B	0.511	0.507	0.122	0.121	0.021	0.020	0.013	0.013	0.015
C	0.296	0.300	0.066	0.067	0.012	0.012	0.012	0.012	0.011
C_v (cal/mol·K)	51.6	55.2	87.0	85.5	163.5	161.7	229.9	224.8	222.1
S (cal/mol·K)	141.1	155.3	197.7	195.1	307.4	304.0	376.5	368.9	355.4
Dipole moment (Debye)	0.044	0.571	0.028	0.463	0.861	0.527	4.377	2.909	8.038

HOMO	-0.357	-0.372	-0.378	-0.390	-0.352	-0.349	-0.253	-0.300	-0.300
LUMO	-0.034	-0.052	-0.093	-0.105	-0.110	-0.122	-0.146	-0.096	-0.121
E_g	0.323	0.320	0.285	0.285	0.242	0.227	0.107	0.204	0.179
IP	0.357	0.372	0.378	0.390	0.352	0.349	0.253	0.300	0.300
EA	0.034	0.052	0.093	0.105	0.110	0.122	0.146	0.096	0.121
χ	0.195	0.212	0.235	0.247	0.231	0.235	0.199	0.198	0.210
η	0.161	0.160	0.142	0.142	0.121	0.113	0.054	0.102	0.089
S	0.081	0.080	0.071	0.071	0.060	0.057	0.027	0.051	0.045
**Ce**	**A-**		**B-**		**C-**		**$Ce(2b)_3$**		
Rotational constants: A (GHz)	0.575	0.582	0.133	0.135	0.023	0.028	0.018	0.018	0.019
B	0.572	0.582	0.129	0.131	0.023	0.020	0.018	0.018	0.017
C	0.328	0.331	0.070	0.071	0.013	0.012	0.011	0.011	0.011
C_v (cal/mol·K)	51.7	53.6	86.8	83.8	163.8	162.4	231.5	225.9	224.1
S (cal/mol·K)	134.3	142.9	188.4	181.6	297.1	296.2	387.8	383.0	379.4
Dipole moment (Debye)	0.098	0.130	0.040	0.036	5.416	2.571	11.523	10.604	5.193
HOMO	-0.553	-0.565	-0.512	-0.513	-0.423	-0.421	-0.342	-0.390	-0.390
LUMO	-0.290	-0.350	-0.288	-0.346	-0.279	-0.337	-0.306	-0.239	-0.296
E_g	-0.263	-0.214	-0.224	-0.167	-0.144	-0.083	-0.107	-0.151	-0.093
IP	0.553	0.565	0.512	0.513	0.423	0.421	0.342	0.390	0.390
EA	0.290	0.350	0.288	0.346	0.279	0.337	0.306	0.239	0.296
χ	0.421	0.457	0.400	0.430	0.351	0.379	0.324	0.315	0.343
η	0.131	0.107	0.112	0.083	0.072	0.042	0.018	0.076	0.047
S	0.066	0.054	0.056	0.042	0.036	0.021	0.009	0.038	0.023
**Pr**	**A-**		**B-**		**C-**		**$Pr(2b)_3$**		
Rotational constants: A (GHz)	0.582	0.575	0.132	0.139	0.026	0.027	0.019	0.018	0.019
B	0.518	0.526	0.123	0.119	0.020	0.019	0.017	0.016	0.016
C	0.314	0.314	0.068	0.070	0.012	0.012	0.010	0.011	0.011
C_v (cal/mol·K)	57.6	55.4	87.3	85.9	163.8	162.2	231.0	225.9	221.9
S (cal/mol·K)	155.4	153.1	190.3	191.5	298.5	303.7	391.2	388.0	375.7
Dipole moment (Debye)	0.233	0.159	0.170	0.294	1.114	1.277	9.726	4.375	3.802
HOMO	-0.358	-0.371	-0.381	-0.391	-0.351	-0.349	-0.252	-0.300	-0.300
LUMO	-0.079	-0.068	-0.091	-0.103	-0.108	-0.122	-0.140	-0.092	-0.107

E_g<br>IP<br>EA<br>χ<br>η<br>S	0.279<br>0.358<br>0.079<br>0.218<br>0.139<br>0.070	0.303<br>0.371<br>0.068<br>0.219<br>0.151<br>0.076	0.290<br>0.381<br>0.091<br>0.236<br>0.145<br>0.072	0.288<br>0.391<br>0.103<br>0.247<br>0.144<br>0.072	0.243<br>0.351<br>0.108<br>0.229<br>0.121<br>0.061	0.227<br>0.349<br>0.122<br>0.235<br>0.113<br>0.057	0.112<br>0.252<br>0.140<br>0.196<br>0.056<br>0.028	0.208<br>0.300<br>0.092<br>0.196<br>0.104<br>0.052	0.193<br>0.300<br>0.107<br>0.203<br>0.096<br>0.048
**Nd**	**A-**		**B-**		**C-**		**$Nd(2b)_3$**		
Rotational constants: A<br>(GHz) B<br>C	0.602<br>0.570<br>0.333	0.658<br>0.533<br>0.335	0.148<br>0.122<br>0.071	0.156<br>0.118<br>0.072	0.023<br>0.023<br>0.013	0.033<br>0.018<br>0.013	0.022<br>0.015<br>0.011	0.022<br>0.016<br>0.011	0.021<br>0.016<br>0.011
C_v (cal/mol·K)<br>S (cal/mol·K)	53.7<br>140.5	53.5<br>140.3	86.7<br>181.7	83.7<br>179.2	161.8<br>283.3	160.3<br>281.6	231.9<br>386.5	224.2<br>371.6	223.8<br>374.1
Dipole moment (Debye)	0.162	0.152	0.958	0.300	5.268	4.161	6.885	7.513	6.175-
HOMO<br>LUMO<br>E_g<br>IP<br>EA<br>χ<br>η<br>S	-0.554<br>-0.325<br>0.229<br>0.554<br>0.325<br>0.439<br>0.114<br>0.057	-0.566<br>-0.341<br>0.225<br>0.566<br>0.341<br>0.453<br>0.112<br>0.056	-0.506<br>-0.329<br>0,177<br>0.506<br>0.329<br>0,417<br>0.088<br>0,044	-0.510<br>-0.338<br>0.172<br>0.510<br>0.338<br>0.424<br>0.086<br>0.043	-0.424<br>-0.315<br>0.109<br>0.424<br>0.315<br>0.369<br>0.054<br>0.027	-0.419<br>-0.331<br>0.088<br>0.419<br>0.331<br>0.375<br>0.044<br>0.022	-0.337<br>-0.316<br>0.021<br>0.337<br>0.316<br>0.326<br>0.010<br>0.005	-0.384<br>-0.277<br>0.107<br>0.384<br>0.277<br>0.330<br>0.054<br>0.027	-0.389<br>-0.289<br>0.100<br>0.389<br>0.289<br>0.339<br>0.050<br>0.025
